# SMYD3 drives cell cycle and epithelial-mesenchymal transition pathways through dual gene transcriptional repression and activation in HPV-negative head and neck cancer

**DOI:** 10.1038/s41598-024-83396-2

**Published:** 2025-01-06

**Authors:** Madhavi Murali, Abbas Saeed, Sohyoung Kim, Kyunghee Burkitt, Hui Cheng, Arfa Moshiri, Jawad Akhtar, Daniel Tsai, Marie Luff, Baktiar Karim, Vassiliki Saloura

**Affiliations:** 1 Thoracic and GI Malignancies Branch, National Institutes of Health, 10 Center Drive, 2B50C, Bethesda, MD 20892 USA; 2 Laboratory of Receptor Biology and Gene Expression, National Cancer Institute, Bethesda, MD USA; 3 National Institute on Deafness and Other Communication Disorders, NIH, Bethesda, MD USA; 4 Molecular Histopathology Laboratory, National Institutes of Health, Frederick, MD USA

**Keywords:** Head and neck cancer, Cancer epigenetics

## Abstract

Human papillomavirus (HPV)-negative head and neck squamous cell carcinoma (HNSCC) is the sixth most common cancer type in the world and is associated with an overall poor prognosis. The protein methyltransferase SET and MYND domain-containing 3 (SMYD3), which trimethylates H3K4, activates gene transcription and enhances several oncogenic pathways, including epithelial-mesenchymal transition and cell cycle related pathways, in various cancer types. It was also recently shown that SMYD3 is overexpressed in HPV-negative HNSCC, and represses the expression of type I IFN response genes, contributing to resistance to anti-PD-1 checkpoint blockade in this disease. In this study, we show that SMYD3 depletion using siRNA interference or CRISPR decreases cellular proliferation and clonal capacity, induces cell cycle arrest and decreases the invasive potential of HPV-negative HNSCC cell lines. Accordingly, xenografts of SMYD3 knockout tumors derived from a human HPV-negative HNSCC cell line grew significantly slower compared to control tumors in mice. Genome-wide mapping for SMYD3 and H3K4me3 in HPV-negative HNSCC cells using cleavage under targets and release using nuclease (CUT&RUN) assays identified direct downstream gene targets regulated by SMYD3, including cell cycle- and EMT-promoting genes. This study provides insights into the epigenetic role of SMYD3 as an oncogene in HPV-negative HNSCC and supports SMYD3 as a rational therapeutic target in HPV-negative HNSCC.

## Introduction

Head and neck squamous cell carcinoma (HNSCC) is the sixth most prevalent non-skin cancer in the world, causing 890,000 new cases and 450,000 deaths in 2018^1^. HNSCC is categorized into two main pathogenetic types: Human-papilloma-virus (HPV)-positive and HPV-negative, which has strong associations with tobacco use. HPV-negative HNSCC is associated with a worse prognosis than its HPV-positive counterpart, and treatment is often multimodal and toxic, with the use of surgical intervention, platinum-based chemotherapy and radiation therapy. Despite this intense multimodality therapy, approximately 50% of patients with HPV-negative HNSCC recur either locoregionally or with distant metastases. Even with the recent advent of immunotherapy, patients with recurrent/metastatic disease have a dismal prognosis, with a median overall survival of approximately 13 months^2^. Based on the above, novel therapeutic approaches are urgently needed.

SET and MYND Domain-containing 3 (SMYD3) is a protein lysine methyltransferase that binds to and activates the transcription of various oncogenes, including epithelial-mesenchymal transition (EMT) and cell cycle-related genes^3–6^. Specifically, Sarris et al.^3^ reported that Smyd3 is necessary for the development of chemically induced liver and colon carcinomas in mice, and that it binds to and activates the transcription of oncogenes through H3K4me3, including EMT genes *Snail1*, *Twist*, *Zeb1*, *Fn1* and *Vimentin*, and cell cycle genes *CcnD1* and *CcnE1*. Aside from its role as an epigenetic regulator, and according to multiple reports showing that protein lysine methyltransferases may have both histone as well as non-histone substrates^7^, a number of cytoplasmic substrates of SMYD3 have been reported. SMYD3 monomethylates MAP3K2 at lysine 260, modulating the PP2A/MAP3K2 interaction and activating the downstream MAPK pathway in K-ras mutant lung and pancreatic adenocarcinoma cell lines^8^. SMYD3 also methylates AKT1 at lysine 14, enhancing the AKT pathway in breast and colorectal cancer cell lines^9^, as well as HER2, enhancing its homodimerization^10^. Furthermore, higher expression of SMYD3 has been associated with poor prognosis in patients with hepatocellular, colorectal and ovarian carcinoma^11^.

Within the context of HPV-negative HNSCC, our group showed that SMYD3 functions as a master epigenetic regulator of immune-related genes and is significantly overexpressed in HPV-negative HNSCC compared to normal and dysplastic buccal epithelium^12^. Specifically, we demonstrated that SMYD3 depletion induced upregulation of type I IFN response and antigen presentation machinery genes in HPV-negative HNSCC cells. SMYD3 repressed immune-related genes via two mechanisms: Through the transcription of *Ubiquitin-like PHD and Ring Finger Domain-containing Protein 1* (*UHRF1*), which encodes for a reader of H3K9me3, a repressive mark found enriched in the promoters of immune-related genes, and by promoting the intragenic deposition of H4K20me3. Importantly, Smyd3 depletion through antisense oligonucleotides increased CD8 + T-cell influx and sensitized an HPV-negative mouse flank tumor model to anti-PD-1 therapy. Accordingly, baseline *SMYD3* mRNA levels predicted pathologic response to neoadjuvant pembrolizumab in HPV-negative HNSCC patients. These data support that SMYD3 functions as a master immunomodulator inducing resistance to immunotherapy in HPV-negative HNSCC.

In this study, we show that SMYD3 also exerts non-immune mediated, direct oncogenic effects in HPV-negative HNSCC cancer cells and tumors. In vitro, SMYD3 depletion decreases cellular proliferation and clonal capacity, induces cell cycle arrest and decreases the invasive potential of HPV-negative HNSCC cells. This is further supported by xenograft mouse models of human HPV-negative HNSCC cell lines, in which SMYD3 knockout (KO) tumors grow at a significantly slower rate compared to control tumors. This is mediated, at least partially, through gene expression changes of specific gene sets occupied by SMYD3. These data further support SMYD3 as a rational therapeutic target in HPV-negative HNSCC.

## Results

### SMYD3 depletion decreases cell proliferation and colony formation capacity of HPV-negative HNSCC cells in vitro and decreases tumor growth in vivo

**Fig. 1:**
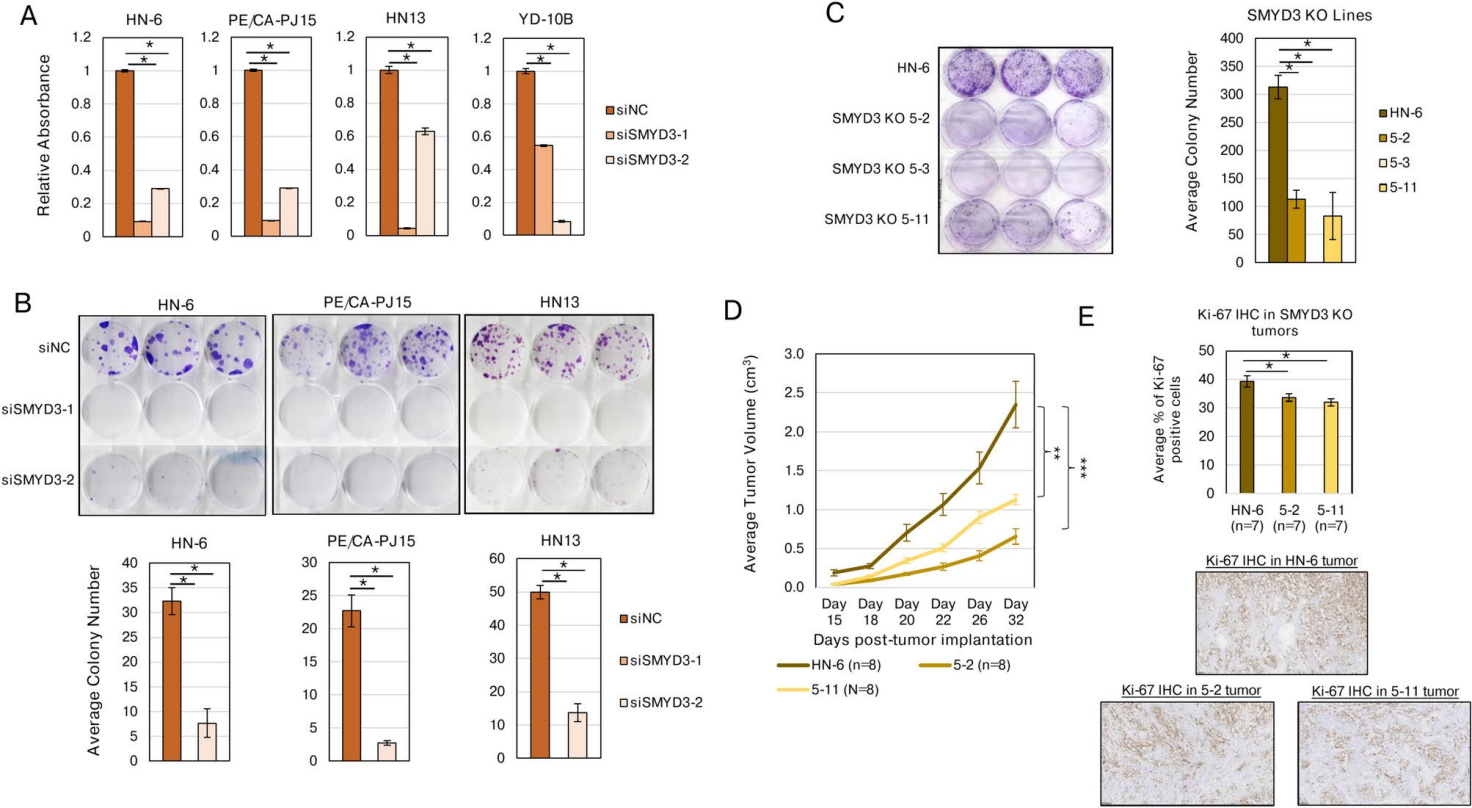
SMYD3 depletion decreases cell proliferation, colony forming capacity and tumor growth in HPV-negative HNSCC cell lines. **(A)** CCK-8 proliferation assays in HN-6, PE/CA-PJ15, HN13 and YD10B. Cells were plated at 5,000 cells/well and were transfected with negative control siRNA or SMYD3 targeting siRNAs (siSMYD3-1, siSMYD3-2). On days 5–7 of incubation following siRNA treatment, CCK-8 was added to the wells on day 5 (YD-10B), day 6 (HN-6, HN13) or day 7 (PE/CA-PJ15) of siRNA transfection and absorbance was measured. Error bars represent six technical replicates per condition. Student t-test, *p-value < 0.05. Standard error bars are shown. This experiment was replicated twice with similar results. **(B)** CFAs in HN-6, PE/CA-PJ15 and HN13 transfected with negative control siRNA or SMYD3 targeting siRNAs (siSMYD3-1, siSMYD3-2). Cells were plated at 500 cells per well in a 6-well plate. Colonies were fixed on days 9–11 following transfection. Colonies were quantified by manual count. Student t-test, *p-value < 0.05. Standard error bars are shown, representing three technical replicates. This experiment was replicated twice with similar results. **(C)** CFAs in HN-6 and SMYD3 CRISPR KO cell lines 5–2, 5–3, and 5–11. Cells were plated at 500 cells per well in a 6-well plate. Cells were fixed and colonies were quantified by manual count. Student t-test, *p-value < 0.05. Standard error bars represent three technical replicates. This experiment was replicated twice with similar results. **(D)** Average tumor volume curves of flank tumors derived from HN-6, 5–2 and 5–11 cell lines implanted in NSG mice. 8 mice were assigned per condition. Student t-test, ** p-value < 0.01, *** p-value < 0.001. Standard error bars are shown. Similar results were obtained in an independent mouse experiment comparing HN-6 and 5–11 tumors. **(E)** Top panel: Bar plot showing the average percentage of Ki-67 positive cells in a tumor section from a representative HN-6, 5–2 and 5–11 tumor sample. IHC for Ki-67 was conducted on FFPE tumor sections. Ki-67 positivity was assessed by QuPath. Bottom panel: Representative images of Ki-67 IHC stain in an HN-6, 5–2 and 5–11 tumor. 200 μm magnification. Student t-test, * p-value < 0.05. Standard error bars are shown.

To assess whether SMYD3 affects cell proliferation in HPV-negative HNSCC cell lines, we conducted CCK8 assays in 4 HPV-negative HNSCC cell lines (HN-6, PE/CA-PJ15, HN13 and YD-10B) after siRNA-mediated knockdown of SMYD3 (Supplementary Fig. 1A). Cells were transfected with negative control and two SMYD3-targeting siRNAs, and CCK8 assays were conducted on days 5–7 of knockdown. siRNA-mediated knockdown of SMYD3 induced a significant decrease in the relative number of viable cells compared to cells transfected with control siRNAs (Fig. 1A).

We next looked into the role of SMYD3 in the colony formation capacity across 3 HPV-negative HNSCC cell lines (HN-6, PE/CA-PJ15, HN13). siRNA-mediated knockdown of SMYD3 induced a significant decrease in the colony formation capacity of all 3 cell lines (Fig. 1B). This phenotype was reproduced in three SMYD3 CRISPR knockout (KO) cell lines (termed 5–2, 5–3, 5–11, Supplementary Fig. 1B), with all SMYD3 KO cell lines showing significant impairment in their colony formation capacity compared to the parental HN-6 cell line (Fig. 1C).

To evaluate whether SMYD3 KO impairs the growth rate of HPV-negative HNSCC cells in vivo in an immunodeficient environment, parental HN-6 cells and two SMYD3 KO cell lines (5–2, 5–11) were implanted in the flank of NSG mice, and tumor growth was assessed. The SMYD3 KO derived tumors exhibited significantly slower growth and had lower Ki-67 protein levels compared to the parental control tumors (Fig. 1D, 1E).

These results support that SMYD3 depletion significantly impairs the proliferative and colony forming capacity of HPV-negative HNSCC cell lines both in vitro and in vivo.

### SMYD3 depletion induces G1 to S phase cell cycle arrest and decreases the migratory and invasive potential of HPV-negative HNSCC cells

**Fig. 2:**
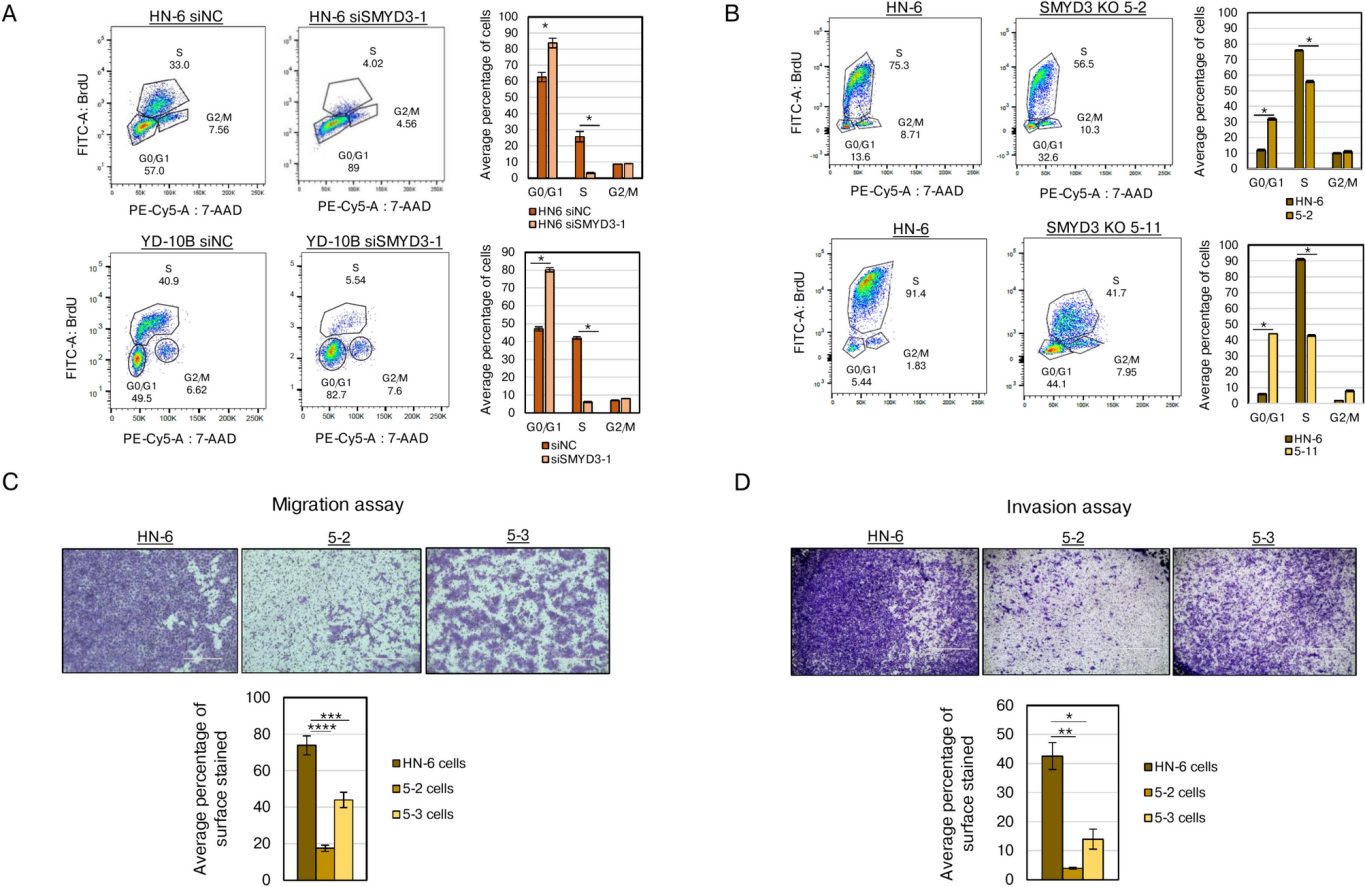
SMYD3 depletion reduces G1/S phase cell cycling and attenuates epithelial to mesenchymal transition in HPV-negative HNSCC cell lines. **(A, B)** BrdU cell cycle analysis of HN-6 and YD-10B cells after 3 days of treatment with control (siNC) or SMYD3-targeting siRNAs **(A)** and of SMYD3 CRISPR KO cell lines 5–2 and 5–11 relative to HN-6 control cells **(B)**. BrdU exposure was performed for 2 h utilizing the BD Pharmigen kit. The left and middle panels show a representative experiment of cell cycle flow cytometry analysis of control (left) and SMYD3 siRNA (middle) treated HN-6 and YD-10B cells. The right panel graphs represent the average of three independent biological replicates for each condition. Student’s t-test, * p-value < 0.05. **(C)** Transwell migration assays with 100,000 cells of HN-6, 5–2, 5–3 SMYD3 KO cell lines in 100uL of DMEM after 22 h of incubation. The graph demonstrates percentage of surface area stained and average of three biological replicates is shown. Student t-test, ***p < 0.001, ****p < 0.0001. **(D)** Transwell invasion assays in HN-6, 5–2 and 5–3 SMYD3 KO cell lines. The graph demonstrates percentage of surface area stained and average of three biological replicates is shown. Student t-test, *p < 0.05, **p < 0.01.

Given that SMYD3 depletion induced a significant proliferative deficit in SMYD3 knockdown and knockout conditions in vitro, we investigated whether SMYD3 affects the cell cycle progression in HPV-negative HNSCC cell lines. Unsynchronized cell cycle flow cytometry was conducted in 2 HPV-negative HNSCC cell lines (HN-6, YD-10B) after transfection with negative control or SMYD3 targeting siRNAs for 3 days. SMYD3 knockdown induced G1 to S phase arrest, with a significant reduction in the S phase and a concordant significant increase in the G1 phase (Fig. 2A). This phenotype was further validated in the CRISPR SMYD3 KO cell lines (Fig. 2B, Supplementary Fig. 2).

Based on previously published work supporting that SMYD3 promotes the epithelial-mesenchymal transition (EMT) phenotype in breast, ovarian and colon carcinoma cells^11^, we sought to determine whether SMYD3 depletion decreases the invasive potential of HPV-negative HNSCC cells. Transwell migration and invasion assays were conducted using the SMYD3 CRISPR KO cell lines 5–2 and 5–3, and the parental HN-6 cell line. SMYD3 KO induced a significant reduction in the migratory and invasive potential of HN-6 cells (Fig. 2C, D) suggesting that SMYD3 promotes the EMT phenotype in HPV-negative HNSCC cells.

The above data support that SMYD3 depletion arrests cell cycle progression and attenuates the invasive potential of HPV-negative HNSCC cells.

### SMYD3 is expressed in both the nuclear and cytoplasmic compartment of HPV-negative HNSCC cell lines

We previously conducted immunohistochemical analysis of SMYD3 and showed that SMYD3 protein expression levels were significantly higher in HPV-negative HNSCC human tumor samples compared to normal squamous epithelium, and expression increased significantly when comparing normal tissue to dysplastic and squamous cell carcinoma tissues^12^. Furthermore, while the pattern of protein expression in cancer cells of HPV-negative HNSCC tumors was predominantly cytoplasmic, nuclear speckles were also observed, supporting the presence of SMYD3 in the nucleus of HPV-negative HNSCC cancer cells^12^.

To further validate the presence of SMYD3 in the nucleus of HPV-negative HNSCC cancer cells, nuclear/cytoplasmic protein extraction in 6 HPV-negative HNSCC cell lines followed by Western blotting for SMYD3 was conducted to compare the protein expression levels of SMYD3 in the nuclear and cytoplasmic compartments across these cell lines. According to our previously reported immunohistochemistry data in human HPV-negative HNSCC tumors^12^, while SMYD3 was predominantly expressed in the cytoplasmic compartment, it was also expressed in the nuclear compartment of HPV-negative HNSCC cell lines (Supplementary Fig. 3). These data support that SMYD3 may exert its oncogenic effects both through nuclear as well as cytoplasmic functions in HPV-negative HNSCC cells. Many cytoplasmic substrates of human SMYD3 have been identified, including HER2, AKT1 and MAP3K2, in various cancer types^8–10^, however, its nuclear function, specifically its genome-wide binding pattern and direct gene targets in human cancer cells remains largely unexplored. Considering this, as well as our recently published work^12^ showing that SMYD3 directly binds to and transcriptionally regulates specific immune-related genes in HPV-negative HNSCC cancer cells, we decided to further focus and dissect the epigenetic function of SMYD3 in HPV-negative HNSCC cancer cells.

### SMYD3 functions both as a transcriptional activator and repressor of specific gene sets in HPV-negative HNSCC cells

**Fig. 3:**
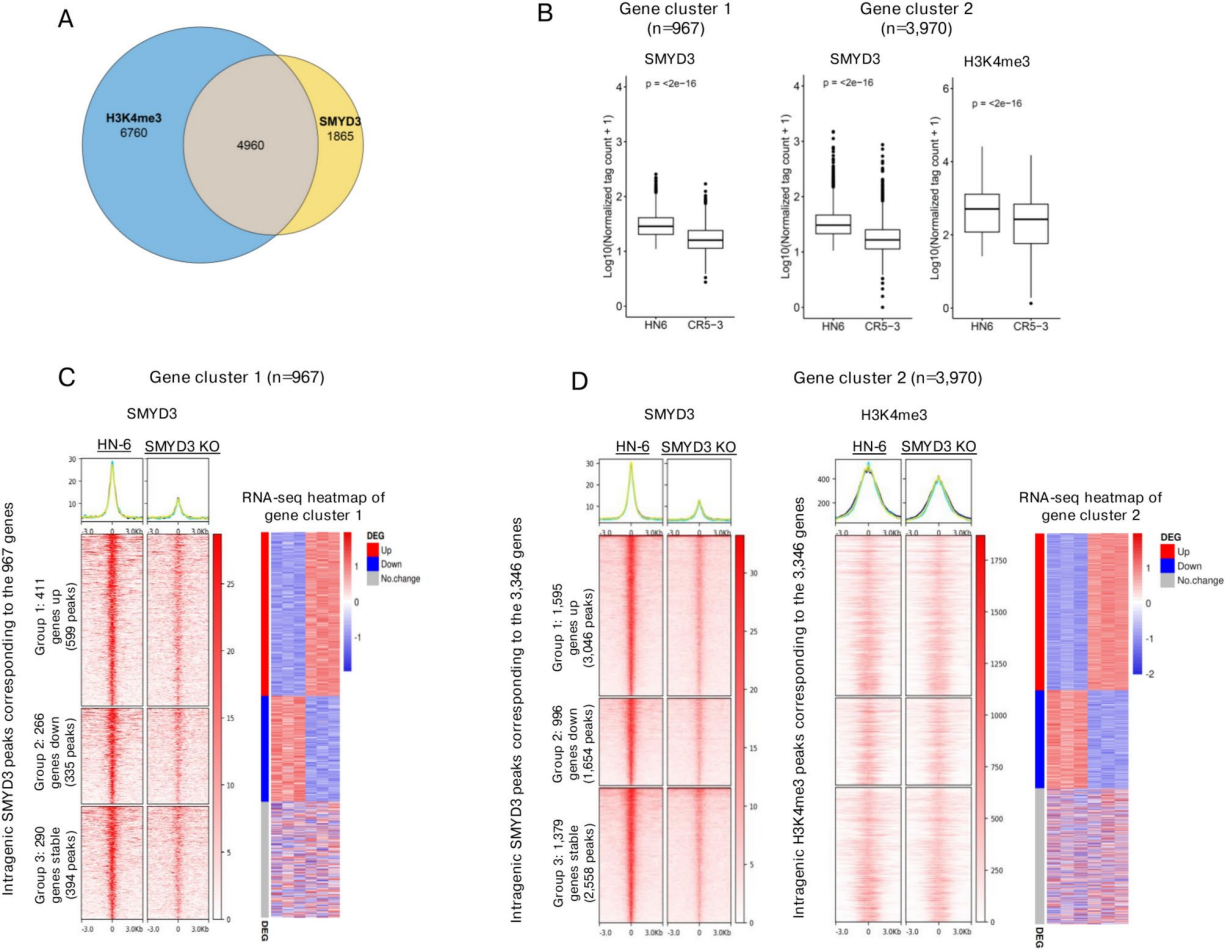
SMYD3 functions both as a transcriptional activator and repressor of specific gene sets in HPV-negative HNSCC cells.** (A)** Venn diagram showing co-ccupancy analysis of genes corresponding to decreased or lost SMYD3 and H3K4me3 intragenic peaks. **(B)** Normalized tag counts (CUT&RUN signal intensity) for SMYD3 (gene cluster 1: n = 1,572 peaks, gene cluster 2: n = 7,258) and H3K4me3 (gene cluster 2: n = 6,418 peaks) at intragenic sites with differentially decreased or lost peaks in HN-6 versus SMYD3 CRISPR KO 5–3 cells. **(C)** Genomic coordinate heatmap of 1,328 lost or decreased SMYD3 peaks corresponding to 967 genes (gene cluster 1), and concordant RNA-seq heatmap. Centered at the peak center, FDR cut off : 0.1, log2FC cut off: 0. **(D)** Genomic coordinate heatmap of 7,258 lost or decreased SMYD3 peaks and 6,418 lost or decreased H3K4me3 peaks corresponding to 3,940 genes (gene cluster 2), and concordant RNA-seq heatmap. Centered at the peak center, FDR cut off : 0.1, log2FC cut off: 0.

SMYD3 has been shown to write and read H3K4me3 in hepatocellular and colon cancer cell lines^3^. To assess whether SMYD3 affects the global levels of H3K4me3 in HPV-negative HNSCC cells, Western blotting for H3K4me3 was conducted in HN-6 cells after 6 days of treatment with SMYD3 targeting siRNAs (Supplementary Fig. 4A). Global levels of H3K4me3 were decreased from ~ 30–100%, supporting that SMYD3 is a major regulator of global H3K4me3 levels in a transient SMYD3 depletion state. To identify direct gene targets transcriptionally regulated by SMYD3 that could provide mechanistic insights into the observed phenotypes of decreased proliferative, colony formation capacity and decreased invasive potential of HPV-negative HNSCC cells after SMYD3 depletion, genome-wide mapping of SMYD3 and its histone enzymatic end-product H3K4me3^3^ was pursued in parental HN-6 and SMYD3 KO cells (5–3 cell line) using CUT&RUN assays. For SMYD3, 16,817 peaks were called in HN-6 cells, with 75% of these occupying intragenic regions and 25% occupying intergenic regions. Of the intragenic peaks, 45% were present in introns, 15% in promoters, 8% in UTR regions and 7% in exons (Supplementary Fig. 5). After SMYD3 KO (5–3 cells), 15,284 peaks were lost or decreased (91%, FDR < 0.1). Of these peaks, 11,452 were intragenic. For H3K4me3, 24,456 peaks were called in HN-6 cells (Supplementary Fig. 5). SMYD3 KO induced significant decrease or loss of 18,385 of these peaks (~ 75%, FDR < 0.1), supporting that SMYD3 is a major regulator of H3K4me3 deposition on chromatin in HN-6 cells. Furthermore, 85% of these peaks were intragenic (15,622), supporting that SMYD3 predominantly affects intragenic H3K4me3 peaks (Supplementary Fig. 5). Interestingly, although the majority of H3K4me3 peaks were significantly decreased or lost after SMYD3 KO, global levels of H3K4me3 remained stable (Supplementary Fig. 4B), suggesting compensatory effects of other methyltransferases on chromatin-unbound H3K4 in the permanent SMYD3 KO state. 6,825 genes were annotated to the 11,452 decreased or lost intragenic SMYD3 peaks and 11,720 genes were annotated to the 15,622 decreased or lost intragenic H3K4me3 peaks (Fig. 3A). Of these genes, 4,960 were co-occupied by SMYD3 and H3K4me3, suggesting a role for SMYD3 and H3K4me3 in the regulation of the transcription of these genes (Supplementary Table 1).

To understand the effect of SMYD3 and H3K4me3 on the transcription of genes, we further focused our analysis on genes with decreased or lost SMYD3 with or without a decrease or loss of H3K4me3 peaks, and available RNA-seq data. Genes occupied by SMYD3 but not H3K4me3 were collectively termed gene cluster 1 (n = 967), and genes co-occupied by SMYD3 and H3K4me3 were collectively termed gene cluster 2 (n = 3,970). As expected, SMYD3 depletion induced a significant decrease in the average normalized tag counts corresponding to SMYD3-binding sites in gene clusters 1 and 2. In gene cluster 2, SMYD3 depletion also induced a decrease in H3K4me3-binding sites, suggesting that SMYD3 promotes the deposition of H3K4me3 on the genes of this cluster (Fig. 3B). To assess the effect of SMYD3 depletion on the transcription of genes in gene clusters 1 and 2, RNA-seq was conducted in SMYD3 KO (5–3) and HN-6 control cells. Of the genes with decreased or lost SMYD3 peaks of gene cluster 1, 43% (411/967) were upregulated (group 1), 28% (266/967) were downregulated (group 2), and 30% (290/967) were unchanged (group 3) (Fig. 3C, Supplementary Table 2). This pattern of expression was maintained in gene cluster 2, where 40% (1,595/3,970) of genes were upregulated (group 1), 25% (996/3,970) were downregulated (group 2), and 35% (1,379/3,970) were unchanged (group 3) (Fig. 3D, Supplementary Table 2).

These data demonstrate that while SMYD3 functions as a transcriptional activator for certain gene sets (group 2, gene clusters 1 and 2), which is concordant with previously reported work^3,4^, it also functions as a repressor for other gene sets (group 1, gene clusters 1 and 2) in HPV-negative HNSCC cells. Interestingly, in the most prevalent group 1 of gene cluster 2, although H3K4me3 occupancy was decreased, transcriptional activity was increased, suggesting that other histone marks may have a more predominant role in regulating the expression of this gene group.

### SMYD3 binds to and regulates the transcription of specific cell cycle- and EMT-related genes in HPV-negative HNSCC cells

**Fig. 4:**
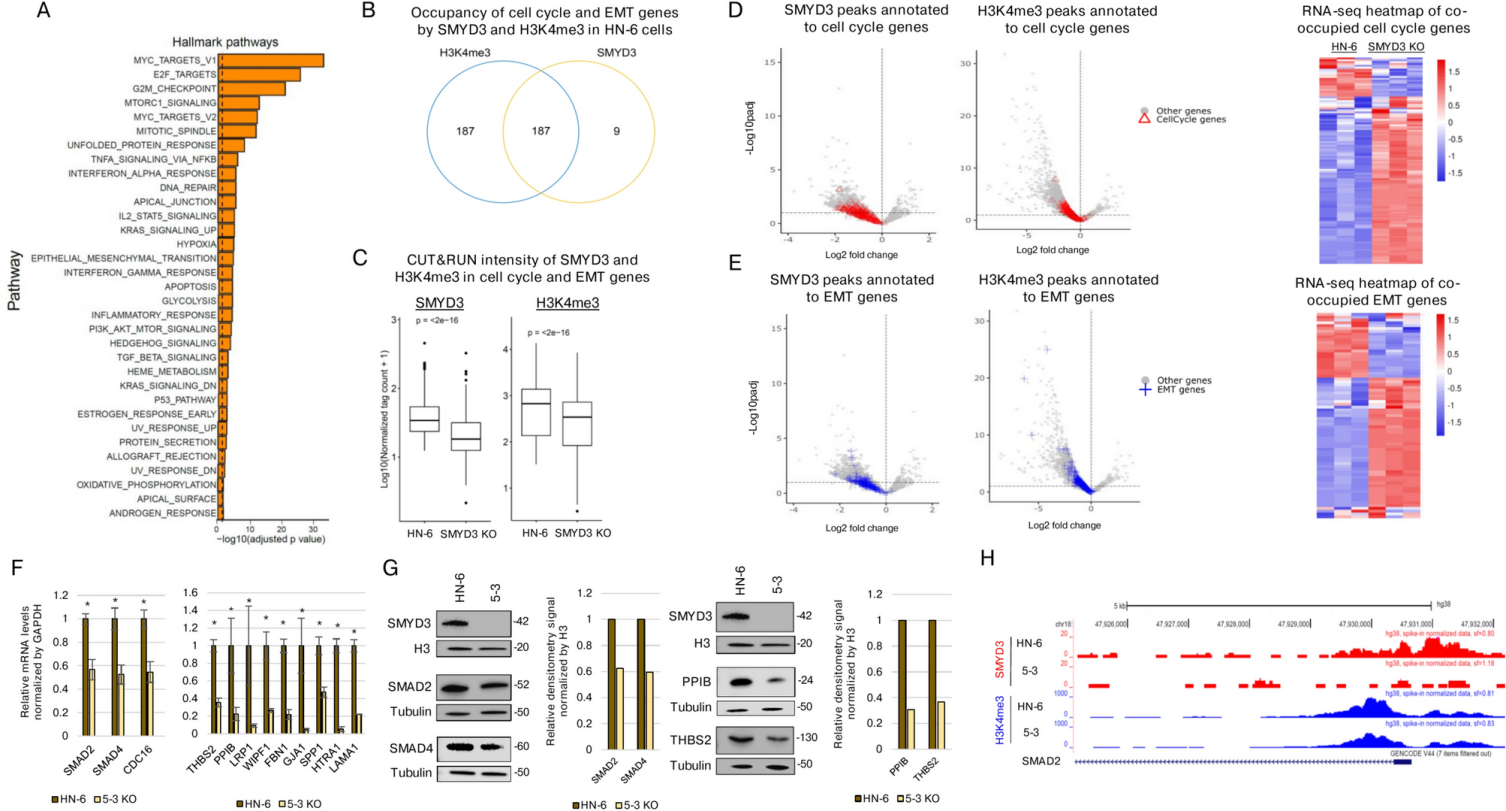
SMYD3 regulates the transcription of specific cell cycle- and EMT-related genes in HPV-negative HNSCC cells.** (A)** Top enriched pathways obtained in GSEA analysis of SMYD3 KO 5–3 vs. HN-6 cells using Hallmark gene sets. GSEA output data were used for the barplot. Adjusted p-value < 0.05. The vertical dotted line corresponds to –log10(padj = 0.05). **(B)** Venn diagram showing the occupancy and co-occupancy of EMT and cell cycle genes by SMYD3 and H3K4me3 (intragenic peaks called in HN-6 cells). EMT and cell cycle genes occupied by SMYD3: 196, genes occupied by H3K4me3: 374, co-occupied genes: 187. **(C)** Left: Boxplot of 408 intragenic SMYD3 peaks called in HN-6 cells, corresponding to 196 EMT and cell cycle genes. Wilcoxon test. Right: Boxplot of 585 intragenic H3K4me3 peaks called in HN-6 cells, corresponding to 376 EMT and cell cycle genes. Wilcoxon test. **(D)** Volcano plot for SMYD3 (left panel) and H3K4me3 (right panel) peaks in SMYD3 KO (5–3 cells) compared to HN-6 cells. Peaks annotated to cell cycle genes are shown as red triangles. Left panel: 3,412 decreased or lost intra- or intergenic SMYD3 peaks were annotated to 2,631 genes. 213 decreased or lost SMYD3 intragenic peaks were annotated to 111 cell cycle genes with available mRNA expression data. Right panel: 17,998 significantly decreased or lost intra- or intergenic H3K4me3 peaks were annotated to 12,495 genes. 208 decreased or lost H3K4me3 peaks were annotated to 111 cell cycle genes. RNA-seq heatmap of co-occupied genes with intragenic SMYD3/H3K4me3 is shown in the far right panel. FDR cut off: 0.1, LFC cut off: 0. Total SMYD3 peaks: 16,993. Total H3K4me3 peaks: 26,441. **(E)** Volcano plot for SMYD3 (left panel) and H3K4me3 (right panel) peaks in SMYD3 KO (5–3 cells) compared to HN-6 cells. Peaks annotated to EMT genes are shown as blue crosses. 176 decreased or lost SMYD3 peaks were annotated to 73 EMT genes with mRNA expression data. 176 decreased or lost H3K4me3 peaks were annotated to 73 EMT genes. RNA-seq heatmap of co-occupied genes with decreased/lost intragenic SMYD3/H3K4me3 peaks is shown in the far right panel. FDR cut off: 0.1, LFC cut off: log2(1.3). **(F)** qPCRs in SMYD3 KO (5–3) and control HN-6 parental cells for cell cycle and EMT genes with decreased or lost SMYD3 and H3K4me3 peaks. Results represent the average + /- SEM of two separate biological replicates with three technical replicates for each sample. Student t-test, * p < 0.05. **(G)** Validating Western blots in representative cell cycle (SMAD2, SMAD4) and EMT genes (PPIB, THBS2) confirming downregulation of the protein levels of these genes in parental HN-6 and 5–3 (SMYD3 KO) cell lines. 10ug of nuclear extracts were loaded for SMYD3 and 20ug of cytoplasmic extracts were loaded for SMAD2, SMAD4, PPIB and THBS2. H3 and tubulin were used as loading controls. Similar results were obtained in biological duplicates. **(H)** Example of UCSC tracks of a representative cell cycle gene (SMAD2) with significantly decreased SMYD3, H3K4me3 and concordant mRNA downregulation.

Given that SMYD3 depletion induced cell cycle arrest and attenuated the EMT phenotype of HPV-negative HNSCC cells, we sought to evaluate whether SMYD3 binds and transcriptionally regulates the expression of cell cycle- and EMT-related genes. Indeed, GSEA analysis of RNA-seq data obtained from the SMYD3 KO 5–3 cell line compared to HN-6 parental cells revealed significant enrichment of cell cycle- and EMT-related Hallmark pathways (Fig. 4A). We then interrogated a GSEA list of cell cycle- and EMT-related genes (Supplementary Table 3) to identify genes with decreased or lost SMYD3 peaks, with or without decreased H3K4me3 peaks. SMYD3 was found to occupy 196 cell cycle and EMT genes, while H3K4me3 was found to occupy 374 cell cycle and EMT genes (Fig. 4B, Supplementary Table 4). Occupancy analysis for SMYD3 and H3K4me3 revealed that the vast majority of cell cycle and EMT genes with decreased or lost SMYD3 peaks (187/196 cell cycle and EMT genes, 95%) were also co-occupied by H3K4me3 (Fig. 4B), as per the genes of gene cluster 2. Additionally, SMYD3 depletion induced a significant decrease in the average normalized tag counts corresponding to SMYD3 and H3K4me3-binding sites within intragenic regions of these cell cycle and EMT genes (Fig. 4C).

Regarding SMYD3, 213 decreased or lost intragenic peaks were mapped to 111 cell cycle genes with available mRNA expression data (Fig. 4D, **left panel**). Of the decreased or lost H3K4me3 intragenic peaks, 208 overlapped with 111 cell cycle genes (Fig. 4D, **middle panel**). For EMT-related genes, 176 SMYD3 peaks mapped to 73 EMT genes were decreased or lost (Fig. 4E, **left panel**). Of the decreased or lost H3K4me3 intragenic peaks, 139 overlapped with 73 EMT genes (Fig. 4E, **middle panel**). Of the 111 cell cycle genes, 61% (68/111) were upregulated, 8% (9/111) were downregulated, and 31% (34/111) were not significantly altered (FDR cut off: 0.1, LFC cut off: 0.38 [log2(1.3)] (Fig. 4D, **right panel**, Supplementary Table 5A). Of the 73 EMT-related genes, 56% (41/73) were upregulated, 22% (16/73) were downregulated and 22% were not significantly altered with SMYD3 KO (FDR cut off: 0.1, LFC cut off: 0.38) (Fig. 4E, **right panel**, Supplementary Table 5B). Some of these cell cycle- and EMT-promoting genes with SMYD3 and H3K4me3 co-occupancy were validated as downstream targets of SMYD3 with qPCR, such as cell cycle-related genes *SMAD family member 2* (*SMAD2*), *SMAD family member 4* (*SMAD4*)*,* and EMT-related genes *Peptidylpropyl isomerase B* (*PPIB*), *Thrombospondin 2* (*THBS2*), *Fibrillin 1* (*FBN1*) and *Laminin Subunit Alpha 1* (*LAMA1*) (Fig. 4F). Downregulation of SMAD2, SMAD4, PPIB and THBS2 was further validated at the protein level (Fig. 4G, Supplementary Fig. 6). Tracks from some of these representative cell cycle- and EMT-related genes are shown in Fig. 4H and Supplementary Fig. 7. Importantly, *Zinc finger E-box binding homeobox 1* (*ZEB1*), a major driver of EMT which was previously reported as a direct gene target of mouse Smyd3 in HCC and colon cancer mouse models^3^, was co-occupied by SMYD3 and H3K4me3 and was significantly downregulated by nearly 60% in the SMYD3 KO state (Supplementary Table 5, Supplementary Fig. 7). *E-cadherin* (*CDH1*), an epithelial marker, was also co-occupied by SMYD3 and H3K4me3 and was significantly upregulated, in accordance with a decrease in the invasive phenotype of HN-6 cells after SMYD3 KO. Interestingly, other EMT-related genes that promote EMT, such as *Vimentin* (*VIM*), *N-cadherin* (*CDH2*), *Snail Family Transcriptional Repressor 1* (*SNAI1*) and *Twist Family BHLH Transcription Factor 1* (*TWIST1*) were also found co-occupied by SMYD3 and H3K4me3, but were significantly upregulated or unaffected (*TWIST1*) in the SMYD3 KO cells despite a decrease in the invasive phenotype of these cells (Supplementary Table 5, Supplementary Fig. 7). The above further support that SMYD3 binds to and functions both as a transcriptional activator for certain EMT-related genes (such as *ZEB1*, *CDH1*, *PPIB*, *THBS2*, *FBN1*, *LAMA1*) and a repressor for other EMT-related genes (such as *VIM*, *CDH2*, *SNAI1*), with the predominant phenotype being that of decreased invasiveness and migration in the SMYD3 KO state of HN-6 cells.

### Cell cycle and EMT regulation pathways are enriched in HPV-negative HNSCC tumors with higher SMYD3 expression

**Fig. 5:**
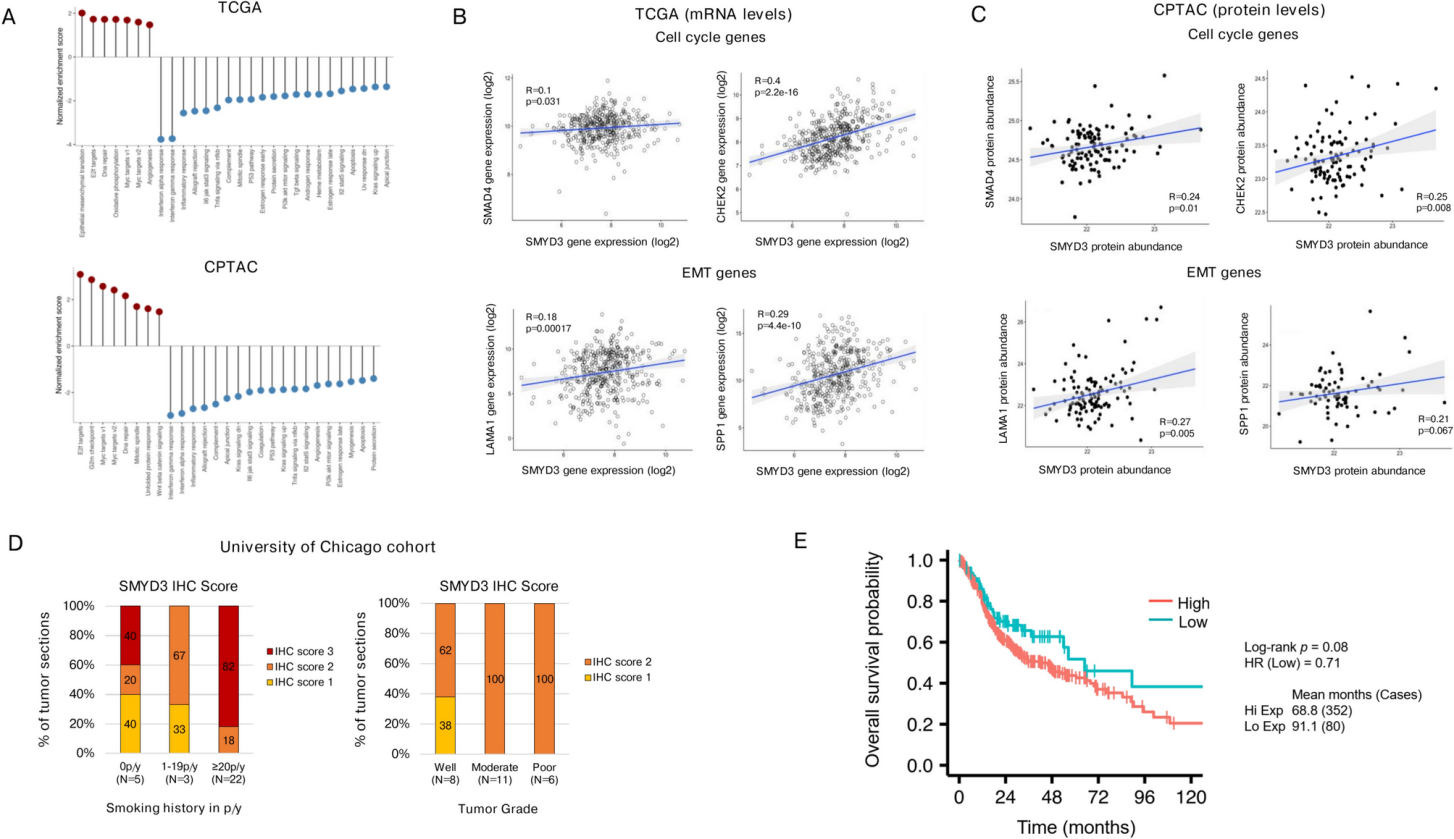
SMYD3 is associated with cell cycle- and EMT-related pathways in patients with HPV-negative HNSCC tumors. **(A) Top:** SMYD3 mRNA expression enrichment analysis in the HPV-negative HNSCC cohort of the TCGA. FDR (q value) < 0.05. **Bottom:** SMYD3 protein level enrichment analysis in the HPV-negative HNSCC cohort of the CPTAC. FDR (q value) < 0.05. **(B, C)** Correlations between log2 transformed mRNA and protein levels of SMYD3 and selected cell cycle and EMT genes in the respective HPV-negative HSNCC cohorts of the TCGA **(B)** and CPTAC **(C)**. Correlation co-efficients R and Wilcoxon p-values shown. **(D)** High SMYD3 protein levels correlate with heavier smoking history (Kruskal–Wallis, p = 0.004) and poor tumor grade (Kruskal–Wallis, p = 0.04). **(E)** Survival Kaplan–Meier curves based on SMYD3 mRNA levels in HPV-negative HNSCC patients. TCGA HPV-negative HNSCC database (n = 483).

We then pursued to interrogate the pathways associated with *SMYD3* mRNA expression in patients with HPV-negative HNSCC tumors. Enrichment analysis of the HPV-negative HNSCC cohort of the TCGA^13,14^ using Hallmark gene sets showed that EMT and cell cycle related pathways (EMT, E2f. targets, Myc targets v1 and v2), as well as DNA repair and angiogenesis were positively correlated with *SMYD3* mRNA levels (Fig. 5A). Conversely, interferon alpha, interferon gamma and inflammatory response pathways were negatively correlated with *SMYD3* mRNA levels, consistently with our previously published work^12^ (Fig. 5A). Similar results were obtained with enrichment analysis utilizing the HPV-negative HNSCC cohort of the CPTAC (Fig. 5A, Supplementary Fig. 8)^15^. These results are in line with the in vitro phenotypes derived with SMYD3 depletion, that is the cell cycle arrest and attenuation of EMT features in HPV-negative HNSCC cells presented above. Furthermore, utilizing the HPV-negative HNSCC cohorts of the TCGA and CPTAC databases, we found that SMYD3 mRNA or protein expression levels correlated positively with select cell cycle and EMT-related genes that we identified as direct downstream targets of SMYD3, which further validates their importance as SMYD3-mediated regulators of the cell cycle and EMT phenotypes in HPV-negative HNSCC (Fig. 5B, C, Supplementary Fig. 9).

### SMYD3 correlates with heavy smoking history and poor tumor grade in HPV-negative HNSCC patients

We previously reported that SMYD3 mRNA and protein levels are significantly increased in HPV-negative HNSCC tumors compared to normal buccal squamous epithelium and that SMYD3 protein expression increases from normal to dysplastic tissues, and then to HNSCC tumors^12^. To assess whether SMYD3 protein levels correlate with clinicopathologic parameters of HPV-negative HNSCC patients, we utilized our previously published, clinically annotated cohort of HPV-negative HNSCC tumors (University of Chicago cohort, n = 39), which includes SMYD3 protein levels quantified by immunohistochemistry of tissue sections as previously described (IHC score scale 0–3)^12^ (Supplementary Table 6). SMYD3 protein levels did not correlate with primary site, stage, tumor size (T), lymph node stage (N), metastatic stage (M), age or gender; however, a significant association was found with both smoking history and tumor grade. Specifically, patients with heavier smoking history (Krustal-Wallis test, p-value = 0.004) and poorly differentiated tumors (Krustal-Wallis test, p-value = 0.04) had significantly higher protein expression levels of SMYD3 (Fig. 5D). Although there was no association of baseline SMYD3 protein levels with overall (OS) and progression-free survival (PFS) in the University of Chicago dataset and the HPV-negative HNSCC cohort of the CPTAC, there was a trend towards worse survival in patients with higher *SMYD3* mRNA levels in the larger HPV-negative HNSCC cohort of the TCGA (OS, log-rank *p* = 0.08, HR = 0.71; PFS, log-rank *p* = 0.07, HR = 0.74) (Fig. 5E, Supplementary Fig. 10).

## Discussion

**Fig. 6:**
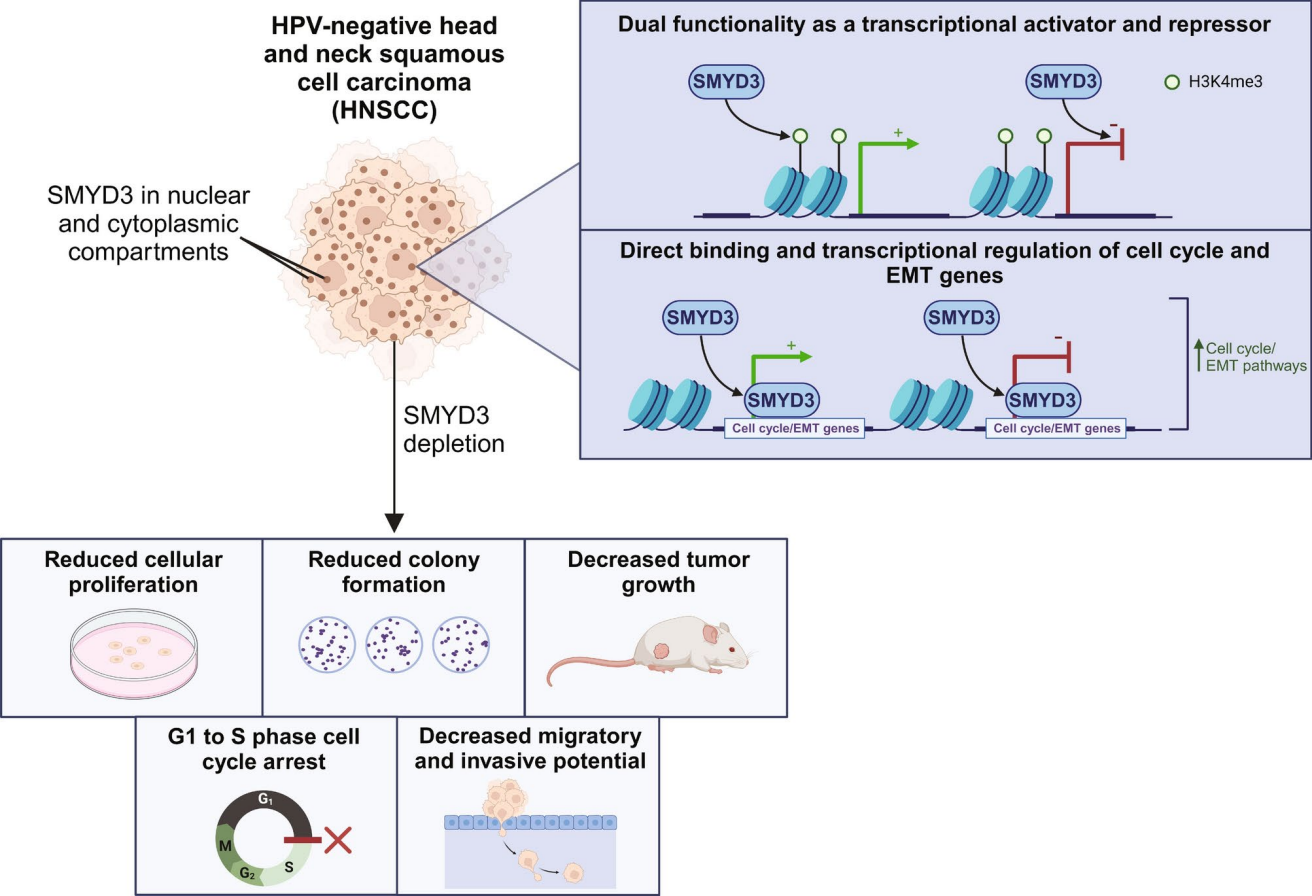
SMYD3 functions both as a transcriptional activator and repressor to promote oncogenic phenotypes in HPV-negative HNSCC cells.

With the current standard of care paradigm, HPV-negative HNSCC patients have an approximately 50% recurrence rate. Despite the advent of immunotherapy, the median overall survival of patients with metastatic disease is still dismal. Thus, identifying novel therapeutic targets for patients with HPV-negative HNSCC is of paramount importance. We have previously shown that the protein methyltransferase SMYD3 represses tumor intrinsic interferon responses in HPV-negative HNSCC through direct transcriptional activation of the H3K9me3-reader UHRF1 which recruits DNMT1A, as well as by promoting the deposition of the repressive mark H4K20me3 on immune-related genes, thus exerting a bifaceted function as a transcription regulator^12^. We also showed that Smyd3 depletion sensitizes HPV-negative HSNCC mouse MOC1 tumors to anti-PD-1 therapy in vivo and that baseline *SMYD3* mRNA expression levels predict response to neoadjuvant pembrolizumab in HPV-negative HNSCC patients. In this work, we show that, independently of its immunomodulatory effects, SMYD3 depletion decreases the proliferative and colony-forming capacity, delays the G1 to S phase transition, decreases the invasive potential of HPV-negative HNSCC cells in vitro, and significantly attenuates tumor growth in vivo in immunocompromised HPV-negative HNSCC mouse models (Fig. 6). Mechanistically, SMYD3 binds to and regulates the transcription of specific cell cycle- and EMT-related genes, such as *SMAD2*, *SMAD4*, *PPIB*, *THBS2*, *FBN1*, *LAMA1*, *ZEB1* and *CDH1*, ultimately promoting the proliferative and invasive potential of HPV-negative HNSCC cells. We also show that higher SMYD3 protein expression levels in human HPV-negative HNSCC tumors are associated with heavier smoking history and poor tumor grade.

Our data support that the observed phenotypes of proliferation, colony formation and invasion are partially mediated through gene expression changes of specific gene sets occupied by SMYD3. We describe two predominant clusters of SMYD3-regulated genes based on their pattern of occupancy by SMYD3 and H3K4me3: Cluster 1 is occupied only by SMYD3, with 43% of the genes upregulated, 28% of its genes downregulated, and 30% unaffected after SMYD3 depletion; cluster 2 is co-occupied by SMYD3 and H3K4me3, with a very similar transcriptional effect as cluster 1 after SMYD3 and H3K4me3 depletion. While SMYD3 is predominantly known to have an activating function on the transcription of its target genes^3,4^, our data suggest its function both as a transcriptional activator and repressor on different gene sets within the same cell context (HPV-negative HNSCC cells). This paradigm of a dual function of a chromatin modifier within the same cell context has been previously reported for Enhancer of Zeste 2 (EZH2), a transcriptional repressor, whereby EZH2 and H3K27Ac, an activating mark, were found to co-occupy the promoter and activate the transcription of the androgen receptor gene in a methyltransferase-independent manner in prostate cancer cell lines^16^. Within the same cell context, EZH2 was also found to co-occupy other gene targets together with H3K27me3, inducing transcriptional repression.

Given the established role of H3K4me3 as an activating histone mark^3^, we expected that depletion of H3K4me3 together with SMYD3 in cluster 2 would result in transcriptional repression as a predominant transcriptional effect. However, the effect was similar to that observed in cluster 1, which was characterized by depletion of SMYD3 but not of H3K4me3. Though the more established function of SMYD3 as a chromatin modifier is that of transcriptional activation through H3K4me3, SMYD3 has been implicated in the transcriptional repression of genes through H4K20me3. Foreman et al.^17^ analyzed and reported the crystal structure of the full length human SMYD3 and, through mutational and biochemical analysis, they identified H4K20 trimethylation as a potential histone substrate for SMYD3. Jiang et al.^18^ reported that SMYD3 binds to and represses the transcription of tumor suppressor *CDKN2A* through H4K20me3, while it also binds to and activates the transcription of *BIRC3* through H3K4me3 in ovarian cancer cell lines. SMYD3 inhibition concordantly led to S phase arrest and increased apoptosis of ovarian cancer cells. More recently, Zeng et al.^19^ demonstrated that SMYD3 binds to and represses the tumor suppressor *epithelial membrane protein 1* (*EMP1*) through H4K20me3 in gastric cancer cells, and that SMYD3 depletion significantly decreases the proliferation of gastric cancer cells in vitro and in vivo. Furthermore, Yang et al.^20^ showed that SMYD3 interacts with the NuRD complex and represses a cohort of genes involved in cell growth and migration, ultimately regulating the proliferation and invasiveness of hepatocellular carcinoma cells (HCC). The authors focused their functional analysis on *insulin like growth factor binding protein 4* (*IGFBP4*) and showed that SMYD3 and the NuRD complex co-occupied the promoter of *IGFBP4*, and that knockdown of SMYD3 led to a decrease in H4K20me3 and a concomitant increase in H4 acetylation of the *IGFBP4* promoter, resulting in transcriptional upregulation of this gene in HCC cells. Finally, our group has also reported a targeted repressive effect of SMYD3 on type I IFN response genes mediated through H4K20me3 in the presence of IFN-β^12^. As such, our findings that SMYD3 functions as an activator and repressor in HPV-negative HNSCC are consistent with previous studies in other cancer types, showing that SMYD3 can act through different mechanisms, including the deposition of activating H3K4me3 and repressive H4K20me3 histone marks, to regulate the transcription of its target genes^12,17–21^. However, whether SMYD3 mediates gene repression through H4K20me3 in HPV-negative HNSCC cells to contribute to the observed phenotypes of proliferation, colony formation and invasiveness remains to be elucidated.

In accordance with the above, we found that SMYD3 binds to specific cell cycle- and EMT-related genes and induces a bifaceted transcriptional output, acting both as an activator in certain genes and repressor on other genes in HPV-negative HNSCC cells (Fig. 4D, E). Importantly, we found that SMYD3 binds to mesenchymal EMT-related genes, such as *PPIB*, *THBS2*, *FBN1*, *LAMA1*, *ZEB1*, and its depletion induced their transcriptional downregulation, supporting that SMYD3 activates the expression of these genes. Interestingly, SMYD3 also binds to the epithelial EMT-related gene *CDH1*, but its depletion induced transcriptional upregulation, supporting that SMYD3 represses the expression of this gene. These findings suggest a coordinated effect towards the promotion of the EMT phenotype through a bifaceted transcriptional function mediated by SMYD3, whereby it acts both as an activator of mesenchymal genes and repressor of epithelial EMT-related genes in HPV-negative HNSCC cells (Fig. 4E). Contrary to our expectation, we also identified commonly reported mesenchymal genes that were transcriptionally upregulated with SMYD3 depletion and thus repressed by SMYD3, such as *VIM*, *CDH2* and *SNAI1*. This could be explained by a recent report supporting the highly complex and context specific nature of the EMT process, whereby certain commonly reported EMT markers may have non-canonical EMT functions depending on the specific cell context^21,22^.

Our study has a number of shortcomings. While our findings suggest that SMYD3 has a bifaceted function both as a transcriptional repressor and activator within the same cell context of HPV-negative HNSCC cells, the mechanism(s) that govern this behavior still remain to be elucidated. One possible explanation, as described above^17–21^ and we previously reported^12^, is that SMYD3 may promote the genome-wide deposition or reading of specific repressive histone marks, such as H4K20me3. This may be mediated through the following mechanisms: (a) SMYD3 directly writes a repressive mark, or activates the methyltransferase activity of another chromatin modifier in an enzymatically dependent or independent manner which in turn writes a repressive mark, such as H4K20me3; (b) SMYD3 activates the transcription of a major transcriptional repressor, as we previously showed for the H3K9me3-reader UHRF1^12^, which then binds to and silences the transcription of target genes; (c) SMYD3 mediates the formation of complexes that repress specific target genes. These possibilities are actively being further investigated by our group. Furthermore, it cannot be excluded that the oncogenic phenotypes of proliferation, colony formation, cell cycling and invasion of HPV-negative HNSCC cells may not only be mediated by the transcriptional functions of SMYD3 in the nucleus of HPV-negative HNSCC cells, but they may also be induced by cytoplasmic functions of SMYD3 through direct methylation of non-histone substrates, as previously reported for other cancer types^8–10^. Additionally, while we pursued to conduct in vitro studies with a number of commercially available SMYD3 inhibitors^23–25^ in order to assess whether the enzymatic activity of SMYD3 is important in the observed phenotypes, the concentrations needed to attain enzymatic inhibition of SMYD3 were inappropriately high in our cell lines, introducing the possibility of non-specific targeting. To address this question, our group is pursuing the generation of enzymatically-inactive SMYD3 expressing HPV-negative HNSCC cell lines. Finally, *SMYD3* mRNA or SMYD3 protein levels were not found to be significantly associated with survival in HPV-negative HNSCC patients; this may be due to the lower number of patients in the examined cohorts, as well as the fact that SMYD3 may be more important in the initial stages of HNSCC carcinogenesis.

In summary, this study demonstrates that SMYD3 depletion significantly impairs the proliferative capacity, cell cycling and the invasive potential of HPV-negative HNSCC cells, while it also hinders tumor growth in vivo. Mechanistically, SMYD3 binds to and regulates the transcription of specific cell cycle and EMT gene sets in HPV-negative HNSCC cells. In addition to our recent report identifying SMYD3 as an epigenetic regulator of immune response in HPV-negative HNSCC^12^, these findings provide further biological rationale to therapeutically target SMYD3 in HPV-negative HNSCC. Furthermore, to our knowledge, this is the first report of genome-wide mapping of human SMYD3 which supports a dual function of this chromatin modifier both as a repressor and an activator of gene expression.

## Methods

### Cell culture

HPV-negative squamous cell carcinoma cell lines HN-6, HN13, PE/CA-PJ15, YD-10B, HN-SCC-151 and SCC-35 cells were derived from patients with locoregionally advanced HPV-negative HNSCC and were kindly provided by Dr. Tanguy Seiwert (University of Chicago). HN-6 and HN13 cells were maintained in DMEM medium with 10% fetal bovine serum, 1% penicillin/streptomycin, and 2 nM L-glutamine. PE/CA-PJ15 cells were maintained IDMEM medium with 10% fetal bovine serum, 1% penicillin/streptomycin, and 2 nM L-glutamine. YD-10B cells were maintained in RPMI medium with 10% fetal bovine serum, 1% penicillin/streptomycin, and 2 nM L-glutamine. HN-SCC-151 and SCC-35 cells were maintained in DMEM/F12 medium, 10% fetal bovine serum, 1% penicillin/streptomycin and 2 nM L-glutamine.

### Generation of SMYD3 knockout cell lines using CRISPR

*SMYD3* CRISPR knockout cell lines (SMYD3 KO 5–2, 5–3, 5–11) were generated from parental HN-6 cells using clustered regularly interspaced short palindromic repeats (CRISPR/Cas9) technology, as previously described^12^. SMYD3 protein expression levels were assessed by Western blotting to confirm the efficiency of the knockout (Supplementary Fig. 1B).

### siRNA transfections

siRNA oligonucleotides were purchased from Millipore-Sigma to target the human *SMYD3* mRNA (SASI_Hs02_0035-5988, termed siSMYD3-1, and SASI_Hs01_0018-8121, termed siSMYD3-2). The negative control siRNA was purchased from Dharmacon (siRNA negative control Dharmacon ON-TARGET plus control pool, #D-001810–10-20, Horizon Discovery, Lafayette, CO). Depending on the assay (CCK8, CFAs, cell cycle flow cytometry), HNSCC cells were plated overnight in 24-well-, 6-well-plates or 10 cm dishes and were transfected with siRNA duplexes (50 nM final concentration) using Lipofectamine RNAimax (Thermo Fisher Scientific, Grand Island, NY) for 3 to 11 days, with re-transfection performed every fourth day.

### CCK8 assays

HNSCC cells were plated overnight in 24-well plates (2–4 × 104 cells/well) and on the next day, they were transfected with SMYD3 (siSMYD3-1 or siSMYD3-2) or control siRNAs (50 nM final concentration) using Lipofectamine RNAimax (Thermo Fisher Scientific) for 5–7 days, with re-transfection performed on day 4. The number of viable cells was measured using the Cell Counting Kit-8 (Dojindo, Kumamoto, Japan) on days 5–7.

### Colony formation assays (CFAs)

HNSCC cells were plated overnight in 6-well plates (500 cells/well) and on the next day, they were transfected with SMYD3 (siSMYD3-1 or siSMYD3-2) or control siRNAs (50 nM final concentration) using Lipofectamine RNAimax (Thermo Fisher Scientific) for 9–11 days, with re-transfection performed on day 4. Once colonies were visible, they were stained with 0.01% (w/v) crystal violet (Sigma-Adrich, cat # HT901-8FOZ), washed with dH20 to remove excess stain, and left to dry. Colonies were counted using the ImageJ software (version 1.53 k).

### Cell cycle analysis with BrdU

Cell cycle analysis was performed using the 5-bromo-20-deoxyuridine (BrdU) flow kit (BD Pharmingen™ FITC BrdU Flow Kit, cat#559,619, BD Biosciences, San Jose, CA) according to the manufacturer’s instructions. Briefly, cells were seeded overnight in 10 cm tissue culture dishes and treated with siSMYD3-1 (50 nM) versus negative control siRNAs (as per above) using medium with 10% FBS for 72 h. 2 h before cell collection, cells were incubated with 10 μM BrdU. Cells were trypsinized, washed, fixed and permealized. Then cells were incubated with DNAase for 1 h at 37 °C, and FITC-conjugated anti-BrdU antibody (dilution: 1:50) was added for 20 min at room temperature. Total DNA was stained with 7-amino-actinomycin D (7-AAD), followed by flow cytometric analysis. Cell cycle analysis was performed in HN-6, YD-10B and HN-5 cells, and in SMYD3 KO cell lines 5–2, 5–3 and 5–11 compared to parental HN-6 cells.

### Transwell migration and invasion assays

For the migration assay, transwell inserts with 8 μm pores (Millicell®, cat #PI8P01250) were placed in a 24-well plate. HN-6 and SMYD3 KO cells were seeded in the upper chambers (100,000 cells/100uL DMEM), and the lower chambers were filled with 600uL of FBS-containing DMEM culture medium. After 24 h of incubation at 37 °C, the upper side of the transwells was gently swabbed with a cotton swab to remove non-migratory cells, while the cells that had migrated through the pores to the lower surface were fixed and stained with 200uL of 0.2% crystal violet for 10-15 min at room temperature. The crystal violet was removed with a cotton swab and by dipping the transwells in dH20. The membranes were left to dry and migrated cells were imaged and counted using ImageJ (version 1.53 k). For the invasion assay, 50uL of Matrigel (Corning™, cat # 354,234) were placed on top of the transwell inserts and was solidified in a 37 °C incubator for 15–30 min to form a thin gel layer before seeding 80,000 cells/100uL DMEM. Following the same 24-h incubation period at 37 °C, the cells that invaded through the Matrigel and transwell pores were similarly fixed and stained with crystal violet, then visualized and counted using ImageJ. Both migration and invasion assays were performed in biological triplicates and were replicated once.

### Western blotting

Nuclear extracts were prepared from HN-6 and CRISPR SMYD3 KO cell lines 5–2 and 5–3 using the Nuclear Complex Co-IP kit (Active Motif) and 10–15 μg were loaded to examine protein levels of SMYD3, with histone H3 used as a loading control. For the detection of cell cycle and epithelial-mesenchymal transition (EMT) regulators, cytoplasmic extracts were prepared using the NE-PER Nuclear and Cytoplasmic Extraction kit (Thermo Scientific, cat # 78,833). 15–20 μg of cytoplasmic protein extracts were loaded to examine protein levels of SMAD2, SMAD4, PPIB and THBS2. Beta-tubulin was used as loading control. To evaluate the abundance of SMYD3 in the nucleus and cytoplasm of HPV-negative HNSCC cell lines, nuclear and cytoplasmic extracts were prepared as per above. 10–15 ug of each extract was loaded for each experiment to examine protein levels of SMYD3. Nuclear extracts were blotted for tubulin and cytoplasmic extracts for histone H3 to confirm the efficacy of nuclear/cytoplasmic separation. Tubulin and histone H3 were used as loading controls for cytoplasmic and nuclear extracts respectively.

Primary antibodies used were anti-SMYD3 (ab187149, Abcam, Cambridge, MA, dilution 1:2000), anti-histone 3 (ab1791, Abcam, Cambridge, MA, dilution 1:125,000), anti-SMAD2 (D43B4, Cell Signaling Technologies, Danvers, MA, dilution 1:1000), anti-SMAD4 (D3R4N, Cell Signaling Technologies, Danvers, MA, dilution 1:1000), anti-PPIB (D1V5J, Cell Signaling Technologies, Danvers, MA, dilution 1:1000), anti-THBS2 (ab84469, Abcam, Cambridge, MA, dilution 1:1000), and anti-beta tubulin (ab6046, Abcam, Cambridge, MA, dilution 1:20,000). Amersham ECL prime Western Blotting Detection Reagent (Cytiva, cat # 45–002-401) or Pierce ECL Western Blotting Substrate (Thermo Scientific, cat# 32,106) were used as detection reagents. Blots were imaged using a Chemi-fluorescent Odyssey FC machine after applying a detection reagent. Densitometry of all blots was performed using ImageJ software (1.53 k, NIH, Bethesda, MD).

### Quantitative real-time PCR

For quantitative real-time PCR, primers for *GAPDH* (housekeeping gene), *SMAD2, SMAD4, CDC16, FOXO4, NDC80, SUGT1, THBS2, PPIB, LRP1, WIPF1, FBN1, GJA1, SPOCK1, SPP1, FBLN5, HTRA1. IGFBP4, LAMA1,* and *LAMA2* were purchased from Sigma-Aldrich. RNA extraction was performed using the RNeasy Mini Kit (cat # 74,004, Qiagen Sciences Inc, Germantown, MD). cDNA conversion was performed using the Invitrogen SuperScript III First-Strand Synthesis System for RT-PCR (cat# 18,080,051, Invitrogen, Carlsbad, CA). PCR was conducted in technical triplicates using SYBR Select Master Mix (cat# 4,472,908, Applied Biosystems, Foster City, CA). PCR reactions were performed using the Applied Biosystems ViiA 7 machine (Thermo Fisher Scientific, Waltham, MA) and the QuantStudio Real-Time PCR Software v1.6.1 (Thermo Fisher Scientific, Waltham, MA). Subsequent analysis of the results was conducted using Microsoft Excel.

### RNA-sequencing

RNA-seq was performed in HN-6 cells and in the CRISPR SMYD3 knockout cell line 5–3. Specifically, parental HN-6 cells and the CRISPR SMYD3 knockout cell line 5–3 were plated, and when cells reached ~ 80% confluence, they were trypsinized, washed twice with PBS, centrifuged and processed for RNA extraction (Direct-zol RNA miniprep kit, Zymo Research, Irvine, CA). Three biological replicates for each sample were processed to extract RNA, quantified using Qubit and sequenced. Samples were pooled and sequenced on NovaSeq Standard_SP using Illumina TruSeq Stranded mRNA Library Prep and paired-end sequencing. The samples had 68 to 130 million pass filter reads. Reads of the samples were trimmed for adapters and low-quality bases using Cutadapt before alignment with the reference genome (hg38) and the annotated transcripts using STAR. The samples had 62–75% non-duplicate reads.

### CUT&RUN assays and DNA-sequencing

For CUT&RUN assays, the 14–1048 CUT&RUN kit by EpiCypher was utilized according to EpiCypher’s protocol. Briefly, CUTANA spike-in dNuc controls (H3K4me0, 1, 2, 3) were mixed together with washed streptavidin (SA) beads in 4 separate 1.5 ml tubes, and incubated for 30 min at RT on nutator. Concanavalin (ConA) beads were activated using cold bead activation buffer, washed twice using a magnet, resuspended in cold activation buffer, added at 10uL/sample in separate strip tubes (1 tube per experimental sample) and kept on ice. 500,000 cells per experimental condition were obtained after trypsinization from respective cell culture dishes (1 dish per biological replicate) and were washed with PBS × 3 to remove excess trypsin. Cells were then resuspended in 100uL/sample of RT wash buffer and washed twice at 600xg for 3 min. After the final wash, cell pellets were resuspended in 105uL of RT wash buffer, and 100uL per sample were aliquoted into each 8-strip tube containing 10uL of activated beads. The cell-bead slurries were incubated on the benchtop for 10 min at RT to allow for adsorption of the cells to the beads. After the incubation, the slurries were placed on a magnet and a small aliquot of the supernatant was obtained to confirm adsorption of cells to the beads (binding efficacy > 93% of cell input). The supernatants were completed removed and the cell-bead slurries were then exposed to cold antibody buffer and vortexed. The CUTANA H3K4MetSTat spike-in control dNucs were added to designated positive (H3K4me3) and negative (IgG) control tubes. Then, 0.5ug of antibody targeting H3K4me3 (EpiCypher, 13–0041) or SMYD3 (Abcam, ab187149) were added to each designated experimental tube. Biological triplicates were used for each experimental condition. The samples were incubated overnight on a nutator at 4 oC. Next day, the 8-strip tubes containing the samples were placed on a magnet till the slurries cleared, supernatants were removed and the cell-beads were washed twice with cold cell permealization buffer. After the final wash, 50uL of the cold cell permealization buffer was added to the cell-bead slurries, and then 2.5uL of pAG-Mnase was added to each sample. Samples were incubated for 10 min at RT and the 8-strip tubes were placed back on a magnet. Supernatants were removed and cell-beads complexes were washed twice with cold cell permealization buffer. After the final wash, 50uL of cell cell permealization buffer was added in each sample, and targeted chromatin digestion followed by adding 1uL of chromatin digest additive to each sample. Strips were incubated for 2 h at 4 oC on a nutator and the reaction was stopped using Stop buffer. 0.5 ng of spike-in Ecoli DNA was added to each sample and samples were incubated for 10 min at 37 oC in a thermocycler. The strips were then placed on a magnet and the supernatants containing the CUT&RUN enriched DNA were transferred to new tubes. DNA was purified per EpiCypher’s protocol, and library construction was conducted. Nucleic acid size selection to enrich for fragment sizes between 200-500 bp was conducted using SPRIselect (Beckman Coulter Life Sciences, B23318). Samples were pooled and sequenced on NextSeq2000 using TruSeq ChIP and Swift Bioscience Accel-NGS 2S Plus DNA Library Prep Kits and paired-end sequencing. All the samples had yields between 53 and 80 million pass filter reads. Samples were trimmed for adapters using Cutadapt before the alignment. The trimmed reads were aligned with hg38 reference using Bowtie2 alignment. All the samples had library complexity with percent non-duplicated reads ranging from 75 to 88%.

### Mouse experiments

All animal experimental protocols, study designs and animal usage were approved and conducted accordingly to all applicable guidelines by the NCI-Bethesda Animal Care and Use Committee and adhering to ARRIVE guidelines. 4–6 week-old female NSG mice were purchased from Jackson Laboratories (stock # 005,557) and used for the described experiments. HN-6, 5–2, and 5–11 cells were grown in vitro and were inoculated by subcutaneous injections of 3 million cells in suspension using Matrigel, in the right flanks of NSG mice. 8 NSG mice were injected per group. Tumor length (L) and width (W) were measured twice weekly with calipers starting from 15 days post-inoculation and tumor volumes were calculated using the formula LxW^2/2. Tumor growth was measured twice weekly starting from 15 days post inoculation. 32 days following tumor inoculation, the mice were euthanized using cervical dislocation, tumors were surgically resected and stained for immunohistochemistry as described below.

### Immunohistochemistry (IHC) for Ki-67

Mouse tumors obtained from NSG mice were stained for Ki-67 using immunohistochemistry (IHC). Briefly, IHC staining was performed on LeicaBiosystems’ BondRX autostainer with the following conditions: Epitope Retrieval 1 (Citrate) 20’, Ki-67 (Cell Signaling Technology #9027, 1:200 incubated 30’), and the Bond Polymer Refine Detection Kit (LeicaBiosystems #DS9800). Isotype control reagent (Cell Signaling Technology #3900) was used in place of the primary antibody as the negative control. Slides were removed from the Bond autostainer, dehydrated through ethanol, cleared with xylenes, and cover-slipped. Images were captured using the Aperio Scanscope FL (Leica Biosystems) whole slide scanner. Image analysis was accomplished using CytoNuclear algorithm in Halo imaging analysis software (v3.5; Indica Labs, Albuquerquee, NM), Image annotations were performed by a pathologist (B.K). Fields were excluded if they contained areas of artifact such as folds or tears.

### IHC of HPV-negative HNSCC tumor samples

As previously described^12^, formalin-fixed, paraffin embedded tissue microarrays containing clinically annotated, de-identified patient tumor samples were obtained from the Human Tissue Research Center of the University of Chicago Pathology Department (IRB#8980). The IHC staining was approved by the Institutional Review Board of the University of Chicago (IRB#18–0468-AM002). 39 HPV-negative HSNCC tumors with clinical annotation, 10 dysplastic lesions and 10 samples from normal buccal epithelium were stained for SMYD3 (Abcam, ab187149) using immunohistochemistry (IHC). The staining was performed on Leica Bond RX automated stainer. After deparaffinization and rehydration, tissue sections were treated with antigen retrieval solution (Leica Microsystems) with heat near 100 °C for 20 min. The anti-SMYD3 antibody (1:400) was applied on tissue sections for 1 h incubation at room temperature. The antigen–antibody binding was detected with Leica Bond Polymer Refine Detection system (Leica Microsystems) and the slides were covered with cover glasses. A head and neck cancer oncologist blinded to clinical outcomes performed semi-quantitative analysis of SMYD3 staining using a four-grade scale defined as follows: Negative, grade 0; mild, grade + 1; moderate, grade + 2; and strong staining intensity, grade + 3. This methodology was chosen based on the observation that SMYD3 staining was observed to be homogeneous in each tissue sample.

### Bioinformatics and statistical analysis

#### Lists of EMT and cell cycle genes

The EMT and cell cycle gene lists were obtained from the Molecular Signature Database (MSigDB) gene sets (Supplementary Table 2, https://www.gsea-msigdb.org/gsea/msigdb/index.jsp) (Fig. 4C, D). Bioinformatic interrogation of these gene lists is shown in Fig. 4. Genes that were not found to be expressed at the mRNA level in the RNA-seq database comparing SMYD3 KO 5–3 with HN-6 parental cells were omitted.

#### RNA-seq heatmaps for EMT and cell cycle genes

RNA-seq data were quantitated to obtain raw tag counts of exon regions at the gene level using featureCount (Fig. 4C and D, right panels). The raw tag count data was variance stabilizing transformed using VST function in DESeq2 R library, and z-score of the transformed data was obtained to color code for heatmap. For clustered heatmaps, pheatmap R library was used with Euclidean distance and ward.D2 clustering options. Significance of gene expression changes was evaluated using DESeq2 R library and determined based on Wald-statistics (FDR < 0.1) and shrunken log2 fold-change (> log2(1.3), < -log2(1.3)) using ahsr method available from DESeq2 library.

#### GSEA analysis of RNA-seq dataset of SMYD3 KO 5–3 and HN-6 parental cell lines

Top enriched pathways (adjusted p-value < 0.05) obtained in Gene Set Enrichment Analysis (GSEA) of SMYD3 KO 5–3 vs. HN-6 cell lines using fgsea (1.22.0) R library and hallmark gene sets (h.all.v7.4.symbols.gmt). GSEA output data were used for the barplot (Fig. 4A).

#### GSEA analysis of TCGA and CPTAC datasets

For the gene-set enrichment analysis (GSEA), 422 TCGA HPV-negative tumor samples (Firehose Legacy) with mRNA expression data (data_RNA_Seq_v2_mRNA_median_Zscores.txt) were analyzed and a ranked gene list was obtained based on Pearson’s correlation of each gene with *SMYD3* expression (Fig. 5A). This list was used as input to the pre-ranked GSEA against the MsigDB’s Hallmark gene sets (h.all.v7.4.symbols.gmt). The GSEA analysis was replicated with protein abundance from CPTAC data portal. Processed proteomics data of 108 HPV(-) HNSCCs were downloaded via LinkedOmics: http://www.linkedomics.org. The specimen collection and sample processing of the tumors can be found in Huang et al. (2021) and the CPTAC data portal (PDC000221, https://pdc.cancer.gov/pdc/study/PDC000221). The GSEA analysis was performed using software GSEA v4.2.3.

#### Correlations between *SMYD3* mRNA or SMYD3 protein levels and candidate downstream target genes

Pearson correlation coefficients were calculated to assess linear associations between *SMYD3* mRNA (TCGA, HPV-negative HSNCC cohort, n = 434) or SMYD3 protein levels (CPTAC, HPV-negative HSNCC cohort, n = 108) and candidate downstream gene target expression levels (Fig. 5B, C). The gene-level proteomics data were represented as normalized mass spectrometric intensity and log2 transformed (Fig. 5B, C).

#### Clinicopathological correlations

SMYD3 protein expression levels were assessed among tumor, dysplasia, and normal samples and clinicopathological correlations were performed using logistic regression models fit to examine the association between IHC score and gender, age, smoking history, stage, T-stage, N-stage and grade (Fig. 5D).

Cox regression was performed to examine whether *SMYD3* mRNA levels (TCGA) or SMYD3 protein levels (CPTAC, Proteomic Data Commons (PDC)) were prognostic for overall or progression-free survival (Fig. 5E, Supplementary Fig. 7). For the survival analysis using the CPTAC HNSCC database (n = 108 patients), protein abundance and clinical data were extracted from the CPTAC data portal (https://proteomics.cancer.gov/data-portal, http://linkedomics.org/data_download/CPTAC-HNSCC). The R function “surv_cutpoint” (package survminer) was applied to identify optimal cutpoints that correspond to the most significant relation with OS and PFS outcomes for SMYD3 protein levels. The data were dichotomized to high and low protein expression groups. The progression-free (PFS) and overall survival (OS) curves were obtained using the Kaplan–Meier (KM) method and were compared using the log-rank test. The Cox proportional hazards model was used to estimate hazard ratios (HRs) with 95% confidence intervals (CIs). Similarly, for the survival analysis using the University of Chicago HPV-negative HNSCC database (n = 35 patients), patients were partitioned into four groups based on dichotomized values of SMYD3 IHC scores derived from the FFPE clinically annotated tissue microarrays.

#### CUT&RUN analysis

Raw fastq files were trimmed with Cutadapt 1.8 and aligned using bowtie2-2.4.4 against hg38. For spike-in controls, the trimmed FASTQ files were aligned against the Escherichia coli MG1655 genome. Duplicated reads were removed using Picard tools 2.26.9, and normalization factors were derived based on the uniquely mapped fragments in the corresponding spike-in control data. The enriched regions with SMYD3 and H3K4me3 signals were identified using GoPeaks spike-in normalization, with default “narrow” parameters applied. Differential analysis was performed using DESeq2 to obtain a final list of regions for downstream analysis (FDR < 0.1). No LFC threshold was applied for CUT&RUN data.

#### Annotation of peaks (CUT&RUN)

Each peak/enriched region in CUT&RUN assays was annotated with the nearby gene displaying the shortest distance between TSS and the center of each peak using the ChipSeeker (1.32.0, GENCODE V39). To account for the EMT and cell cycle genes missed by the ChIP-Seeker program at their default parameters, we used genomic coordinates of gene boundaries to identify additional EMT and cell cycle genes overlapping with CUT&RUN peaks either at their gene body or promoter region (< 2 kb from upstream boundary). The coordinates of gene boundaries of EMT and cell cycle genes (Supplementary Table 2) were extracted by BioMart (v. 2.54.0) R library using Ensembl GRCh38.p13 database. Then GenomicRanges (v.1.48.0) R library was used to find overlapping CUT&RUN peaks and genes based on their extended gene boundary.

#### Volcano plots

For all volcano plots, EnhancedVolcano (1.14.0) R library was used.

#### Statistical analyses of in vitro, in vivo assays and Ki-67 IHC results

Averages of at least three biological replicates from each control and experimental condition were obtained for MTT assays, CFAs, flow cytometry, invasion and migration assays. For the in vivo mouse experiments, averages of tumor volumes were obtained from 8 mice per group. For the Ki-67% positive cells, the average was obtained from 7 out of 8 tumors of each mouse group. One mouse per group was excluded from the Ki-67 level evaluation as they were considered significant outliers compared to the rest of the Ki-67% positive values. Student t-test was used to conduct statistical evaluations between control and experimental conditions.

## Supplementary Information

Supplementary Information 1.

Supplementary Information 2.

Supplementary Information 3.

Supplementary Information 4.

Supplementary Information 5.

Supplementary Information 6.

Supplementary Information 7.

## Data Availability

Data is provided within the manuscript or supplementary information files. Data is provided within the manuscript or supplementary information files. All sequencing raw and processed data have been deposited in the Gene Expression Omnibus (GEO) database and are publicly available under the series GEO: GSE280251 and GSE280252.
