# Supplementary material for: SMYD3 drives cell cycle and epithelial-mesenchymal transition pathways through dual gene transcriptional repression and activation in HPV-negative head and neck cancer
Source: Sci Rep. 2025 Jan 6;15:984. doi: 10.1038/s41598-024-83396-2 (PMC11704228; doi:10.1038/s41598-024-83396-2)
Supplement: Supplementary file 1 — Supplementary Information 1. [file 41598_2024_83396_MOESM1_ESM.pdf]

Supplementary Figure 1. Western blotting for SMYD3 in nuclear extracts obtained from **(A)** four representative HPV-negative HNSCC cell lines, HN-6, HN13, YD-10B and PE/CA-PJ15 treated with two SMYD3-targeting siRNAs (siSMYD3-1, siSMYD3-2) for 3 days, and **(B)** HN-6 and three SMYD3 CRISPR KO cell lines 5-2, 5-3 and 5-11. 10-20ug of nuclear extracts were loaded. H3 was used as a control.

(A)

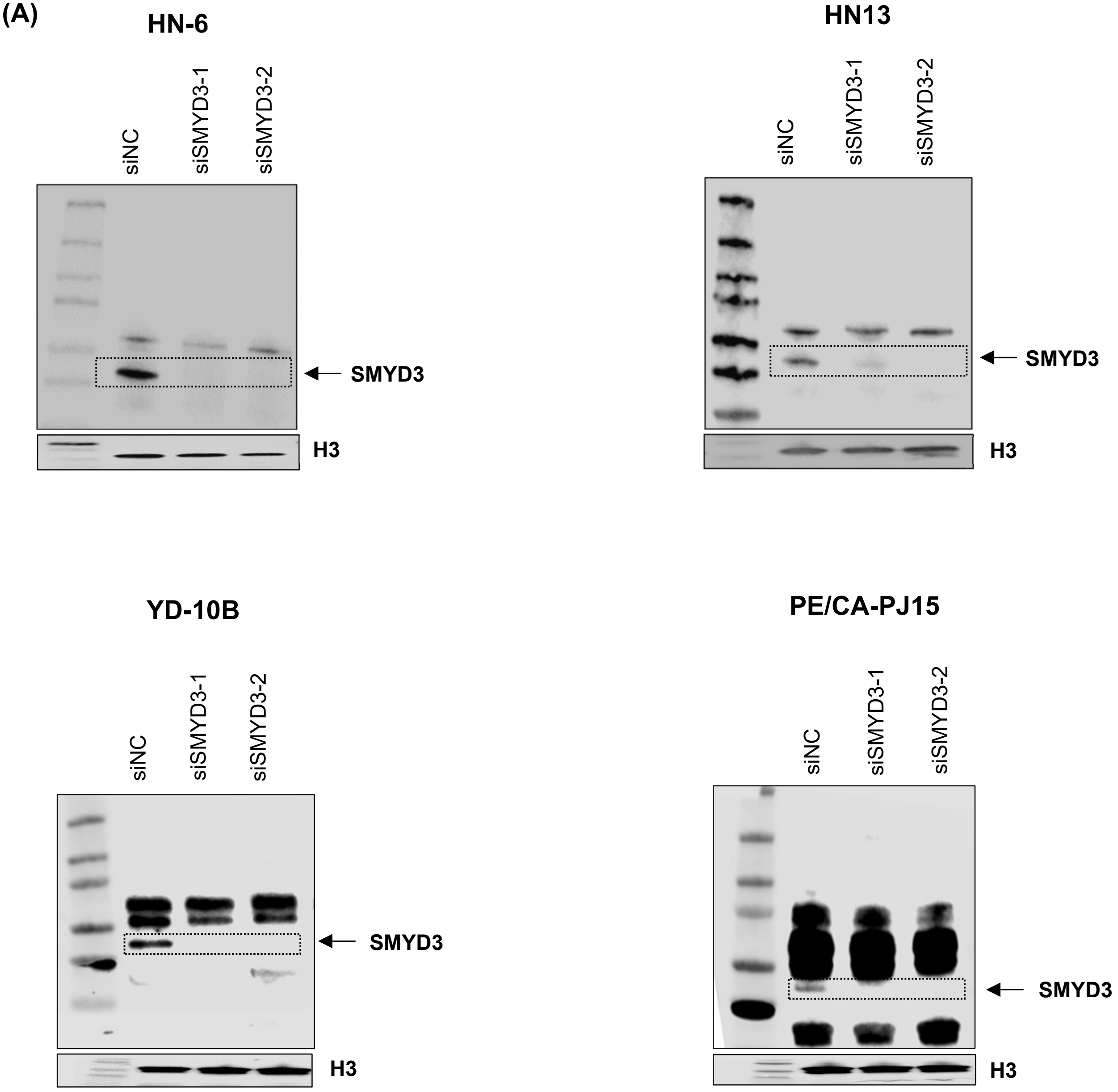

(B)

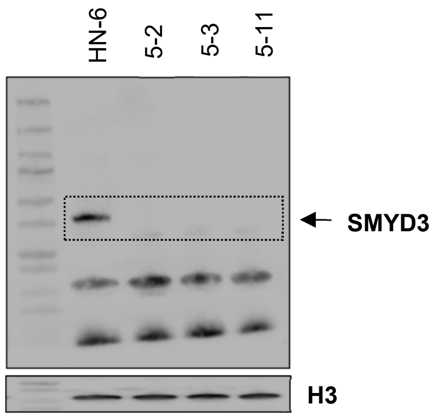

Supplementary Figure 2. Cell cycle flow cytometry after permanent SMYD3 knockout (CRISPR). SMYD3 KO cells (5-3) were compared to parental HN-6 cells. The left and middle panels show a representative experiment of cell cycle flow cytometry analysis of control (left) and SMYD3 siRNA (middle) treated HN-5 or SMYD3 KO 5-3 cells. The right graphs represent the average of three biological replicates. Standard errors are shown. Student t-test, *p<0.05.

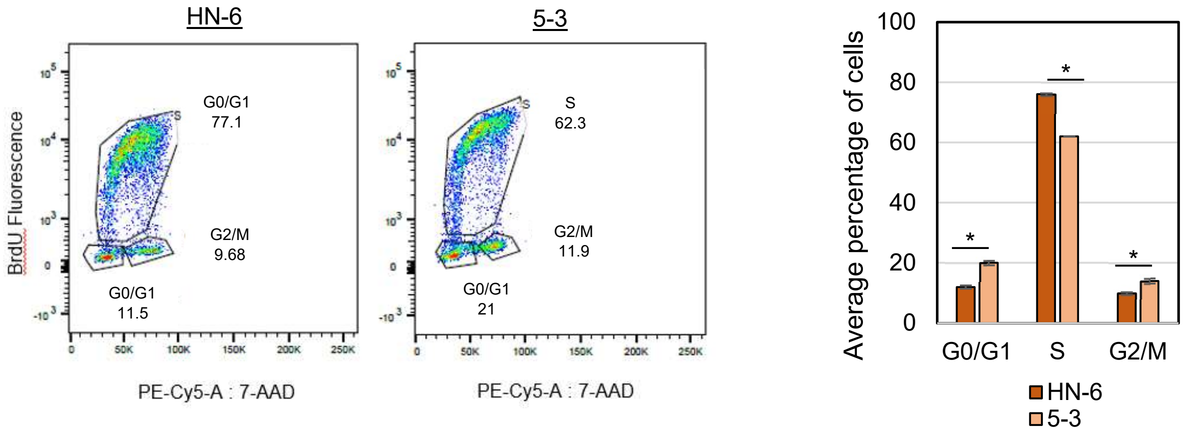

Supplementary Figure 3. Western blotting for SMYD3 in nuclear and cytoplasmic extracts from 6 HPV-negative HNSCC cell lines. 10ug of nuclear or cytoplasmic extracts were loaded per cell line. H3 and beta-tubulin were used as loading controls.

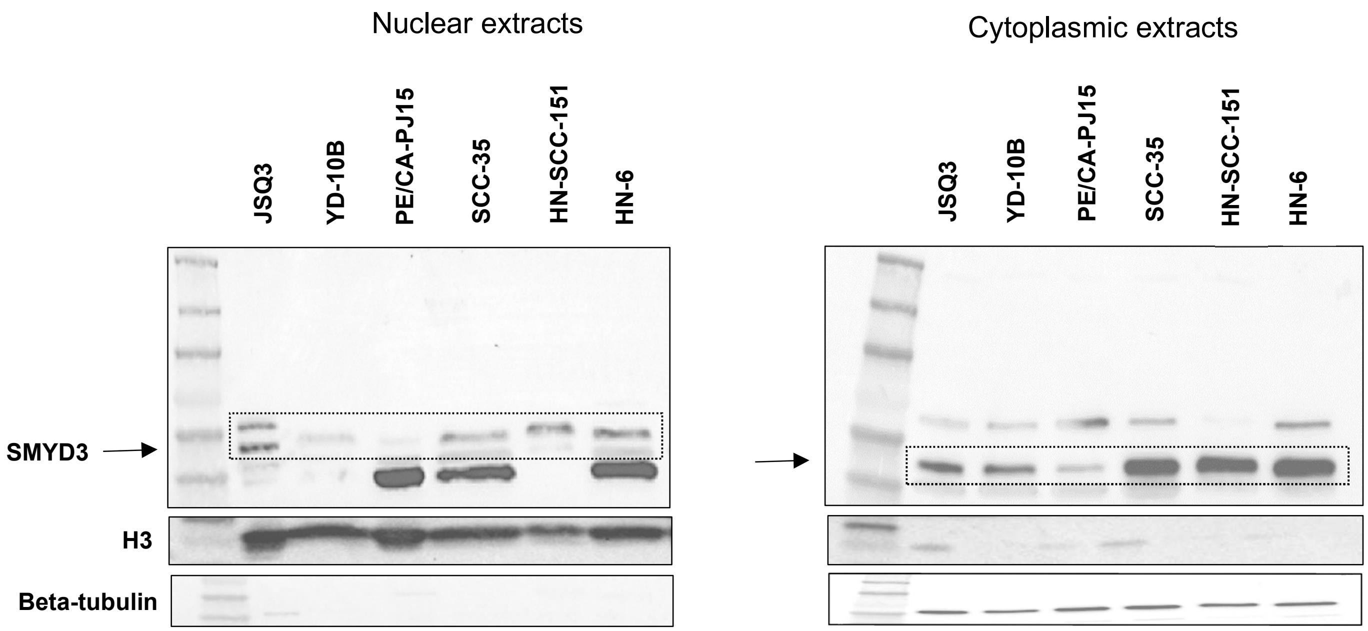

Supplementary Figure 4. Western blotting for H3K4me3 in nuclear extracts obtained from **(A)** HN-6 cells treated with negative control (siNC) or two SMYD3 targeting siRNAs (siSMYD3-1, siSMYD3-2). HN-6 cells were treated with siRNAs for 6 days. Results from two biological replicates are shown. Densitometry results are provided for each replicate in the graphs below each blot. **(B)** HN-6 and three SMYD3 CRISPR KO cell lines 5-2, 5-3 and 5-11. 5ug of nuclear extracts were loaded. H3 was used as a control.

(A)

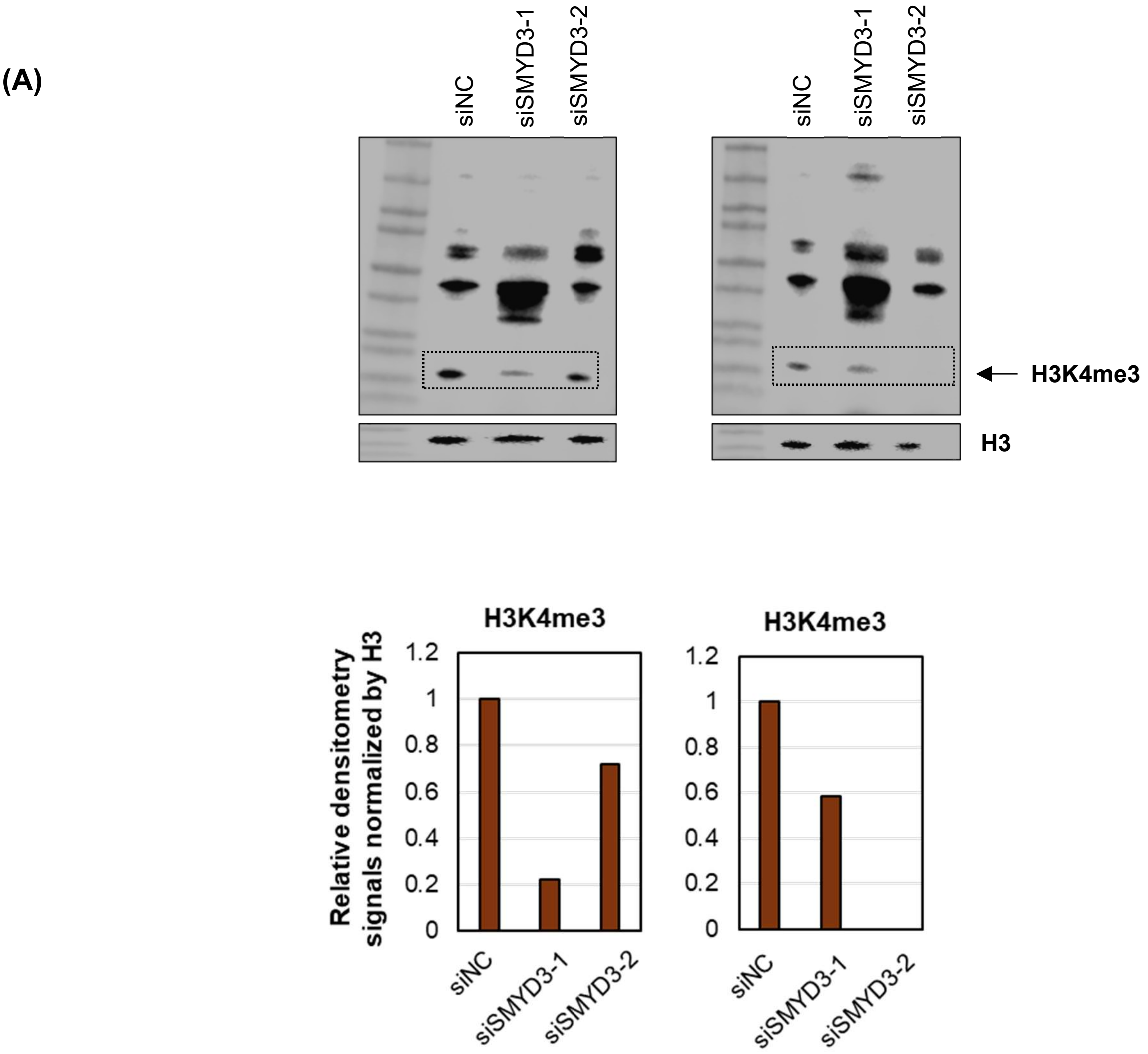

(B)

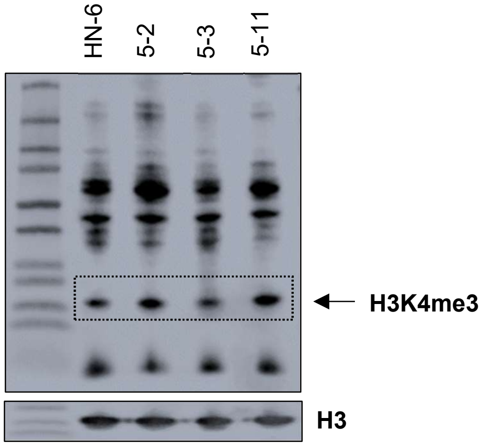

Supplementary Figure 5. Top pie charts: Genome-wide distribution of SMYD3 **(left)** and H3K4me3 **(right)** peaks in HN-6 cells. **Bottom pie charts:** Genome-wide distribution of significantly decreased or lost SMYD3 **(left)** and H3K4me3 **(right)** peaks after SMYD3 KO. 16,817 SMYD3 peaks and 24,456 H3K4me3 peaks were called in HN-6 cells (narrow GoPeaks). 15,284 SMYD3 peaks were lost or significantly decreased after SMYD3 KO in 5-3 compared to HN-6 cells. 75% of these peaks were intragenic (11,425) and were mapped to 6,825 genes. 18,385 H3K4me3 peaks were lost or significantly decreased after SMYD3 KO in 5-3 compared to HN-6 cells. 85% of these peaks were intragenic (15,622) and were mapped to 11,720 genes.

Genomic distribution of SMYD3 in HN-6 cells (N=16,817)

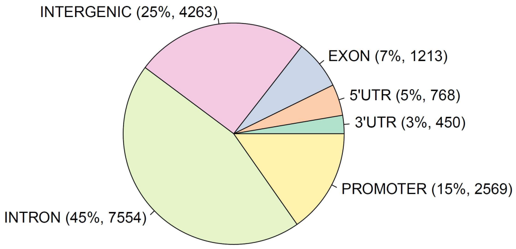

Genomic distribution of H3K4me3 in HN-6 cells (N=24,456)

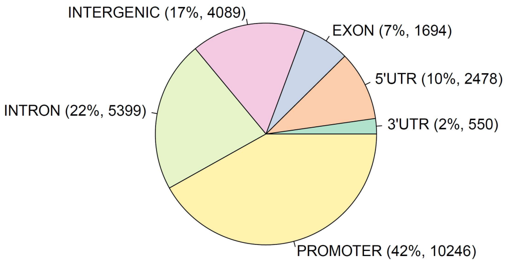

Genomic distribution of lost or decreased SMYD3 peaks after SMYD3 KO (N=15,284)

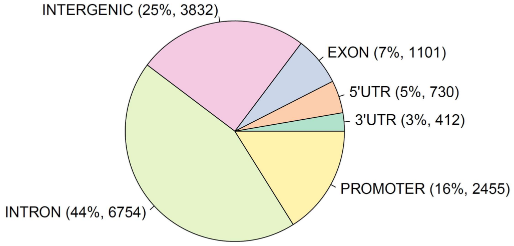

Genomic distribution of lost or decreased H3K4me3 peaks after SMYD3 KO (N=18,385)

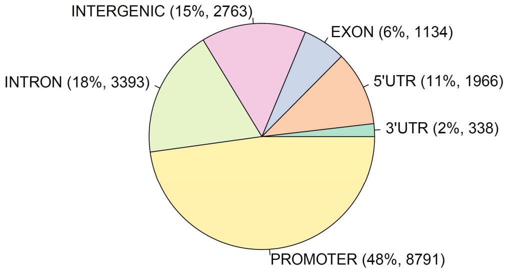

Supplementary Figure 6. Full length blots for Figure 4G. **(A)** SMAD2 (~52kD) and loading control tubulin, **(B)** SMAD4 (~60kD) and loading control tubulin, **(C)** PPIB (~24kD) and loading control tubulin, **(D)** THBS2 (~130kD) and loading control tubulin. Cytoplasmic extracts were obtained from parental HN-6 and SMYD3 KO 5-3 cell lines. 20ug of cytoplasmic extracts were loaded for SMAD2, SMAD4, PPIB and THBS2. The dashed rectangle shape delineates the part of the blot cropped and shown in Figure 4G. Two biological replicates are shown.

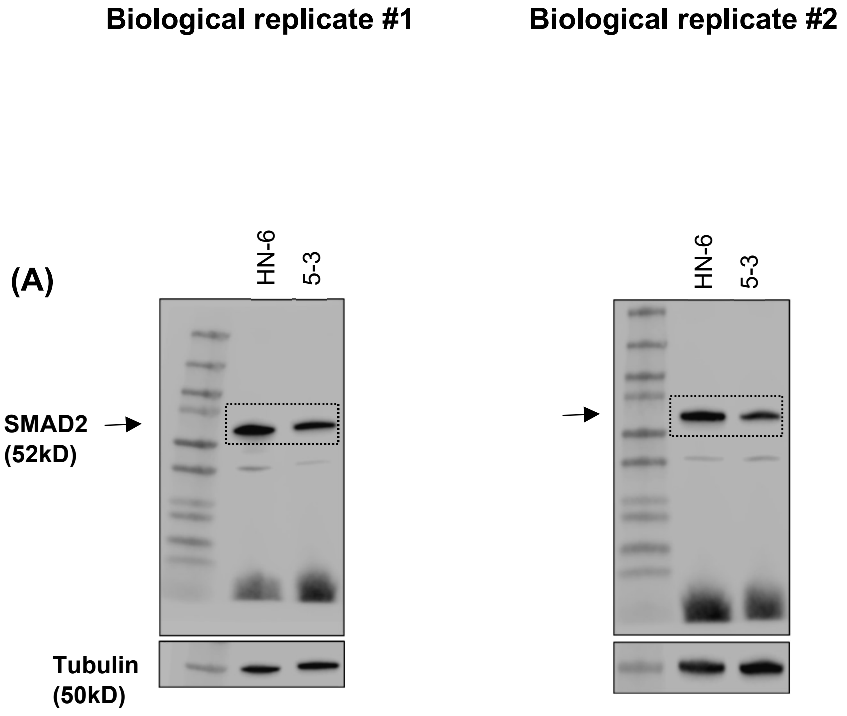

(B)

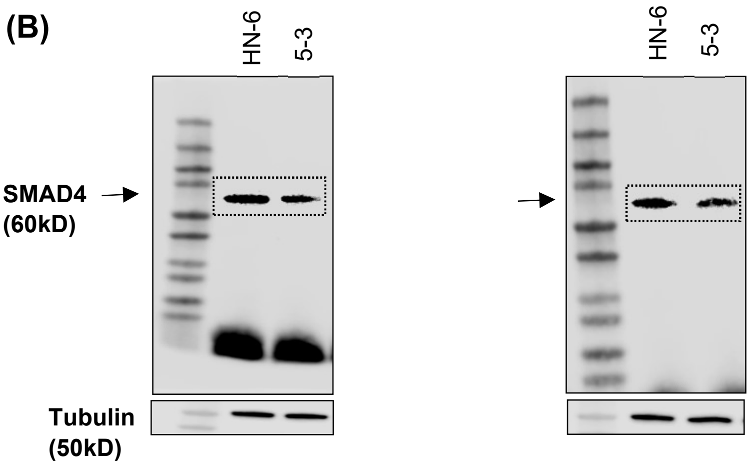

HN-6
5-3
SMAD4
(60kD)
Tubulin
(50kD)
HN-6
5-3

(C)

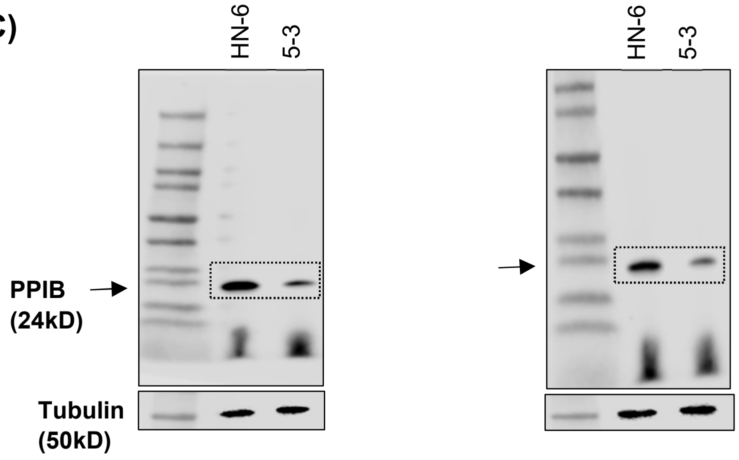

HN-6
5-3
PPIB
(24kD)
Tubulin
(50kD)
HN-6
5-3

(D)

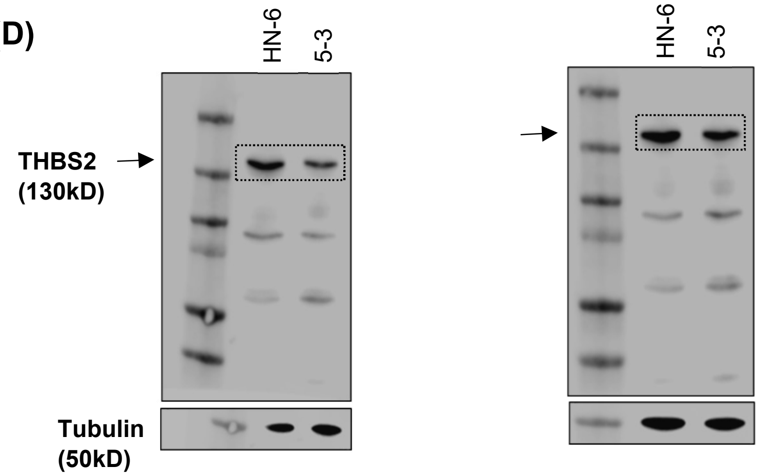

HN-6
5-3
THBS2
(130kD)
Tubulin
(50kD)
HN-6
5-3

Supplementary Figure 7. USCS tracks of CUT&RUN for SMYD3 and H3K4me3, and RNA-seq tracks in SMYD3 KO 5-3 and HN-6 parental cells. Tracks for the cell-cycle related gene *SMAD4*, and for the EMT-related genes *PPIB*, *THBS2*, *ZEB1*, *CDH1*, *CDH2*, *VIM*, *TWIST1* and *SNAI1* are shown. SMYD3 tracks are shown in red, H3K4me3 tracks in blue and RNA-seq tracks in pink and black.

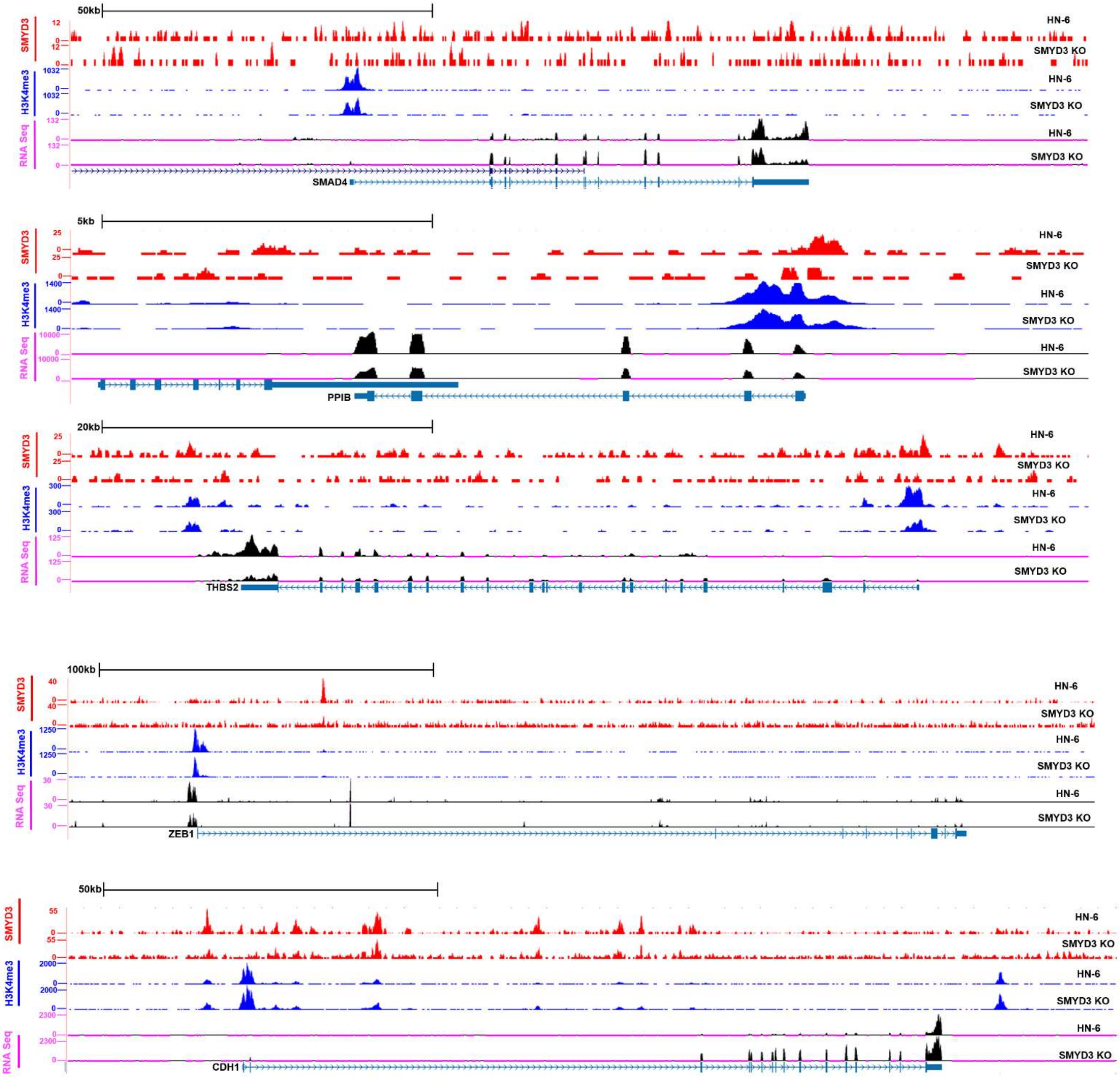

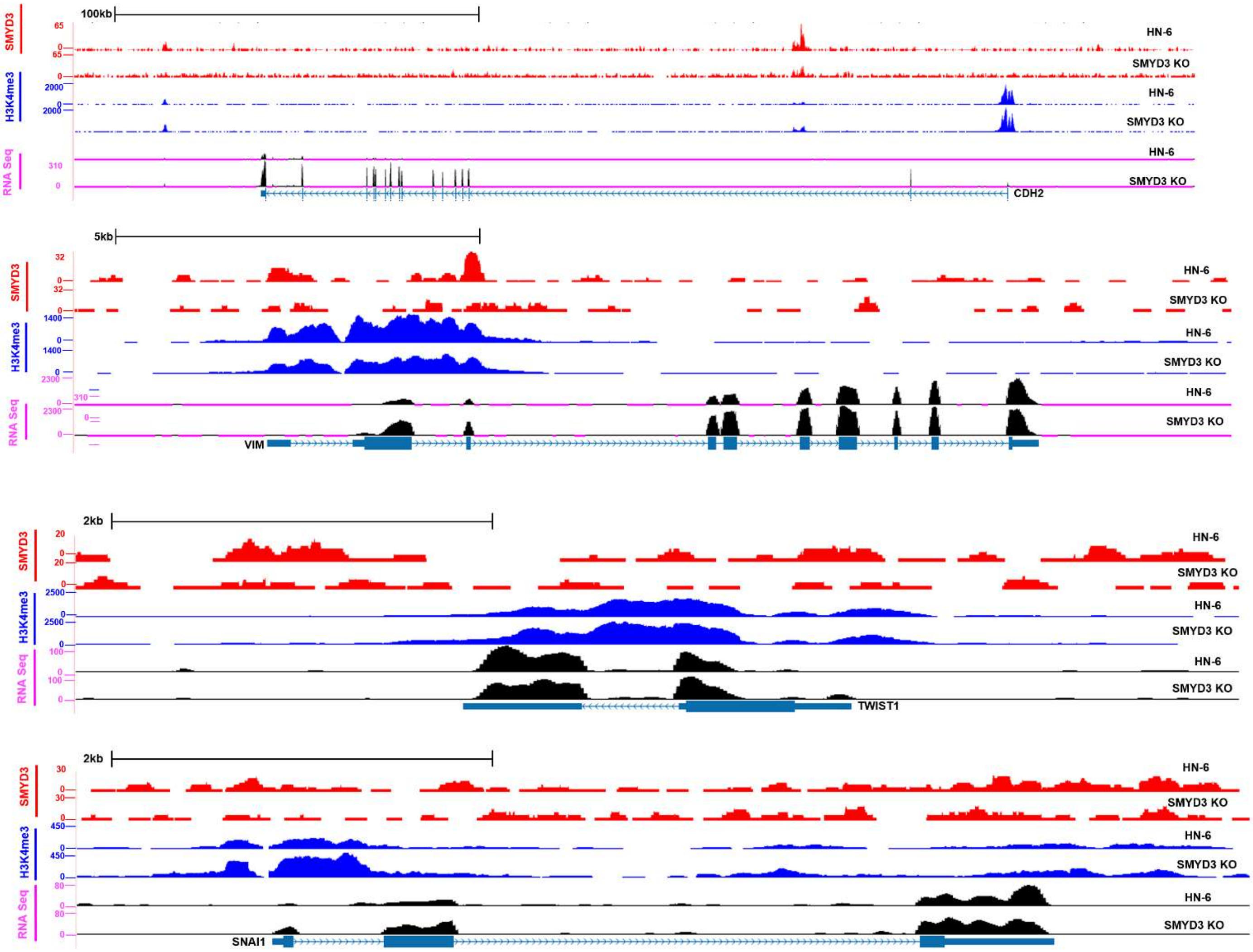

100kb
SMYD3
H3K4me3
RNA Seq
65
0
2000
310
HN-6
SMYD3 KO
CDH2
5kb
32
1400
2300
VIM
2kb
20
2500
100
TWIST1
2kb
30
450
80
SNAI1

Supplementary Figure 8. GSEA analysis for *SMYD3* mRNA in the HPV-negative cohort of the CPTAC.

Table: Gene sets enriched in phenotype na [plain text format]

	GS<br>follow link to MSigDB	GS DETAILS	SIZE	ES	NES	NOM p-val	FDR q-val	FWER p-val	RANK AT MAX	LEADING EDGE
1	HALLMARK_E2F_TARGETS	Details ...	173	0.67	3.08	0.000	0.000	0.000	2386	tags=75%, list=25%, signal=97%
2	HALLMARK_G2M_CHECKPOINT	Details ...	155	0.62	2.85	0.000	0.000	0.000	2517	tags=68%, list=26%, signal=90%
3	HALLMARK_MYC_TARGETS_V1	Details ...	190	0.55	2.57	0.000	0.000	0.000	1970	tags=47%, list=20%, signal=58%
4	HALLMARK_MYC_TARGETS_V2	Details ...	50	0.63	2.41	0.000	0.000	0.000	1794	tags=64%, list=19%, signal=78%
5	HALLMARK_DNA_REPAIR	Details ...	130	0.49	2.16	0.000	0.000	0.000	1973	tags=42%, list=20%, signal=51%
6	HALLMARK_MITOTIC_SPINDLE	Details ...	181	0.36	1.70	0.000	0.005	0.048	1842	tags=29%, list=19%, signal=35%
7	HALLMARK_UNFOLDED_PROTEIN_RESPONSE	Details ...	90	0.38	1.61	0.004	0.014	0.144	2254	tags=34%, list=23%, signal=45%
8	HALLMARK_WNT_BETA_CATENIN_SIGNALING	Details ...	22	0.47	1.47	0.054	0.049	0.479	2171	tags=45%, list=22%, signal=58%
9	HALLMARK_MTORC1_SIGNALING	Details ...	166	0.29	1.33	0.031	0.163	0.915	3384	tags=52%, list=35%, signal=78%
10	HALLMARK_SPERMATOGENESIS	Details ...	55	0.30	1.15	0.223	0.487	1.000	2225	tags=36%, list=23%, signal=47%
11	HALLMARK_NOTCH_SIGNALING	Details ...	21	0.36	1.11	0.309	0.564	1.000	1828	tags=38%, list=19%, signal=47%
12	HALLMARK_HEDGEHOG_SIGNALING	Details ...	23	0.35	1.09	0.362	0.578	1.000	1665	tags=30%, list=17%, signal=37%
13	HALLMARK_PANCREAS_BETA_CELLS	Details ...	15	0.39	1.09	0.338	0.534	1.000	656	tags=20%, list=7%, signal=21%
14	HALLMARK_UV_RESPONSE_UP	Details ...	113	0.25	1.07	0.328	0.545	1.000	1692	tags=25%, list=18%, signal=30%
15	HALLMARK_PEROXISOME	Details ...	86	0.24	1.00	0.455	0.692	1.000	2016	tags=23%, list=21%, signal=29%
16	HALLMARK_GLYCOLYSIS	Details ...	159	0.21	0.98	0.518	0.713	1.000	2431	tags=28%, list=25%, signal=37%
17	HALLMARK_REACTIVE_OXYGEN_SPECIES_PATHWAY	Details ...	43	0.23	0.86	0.709	1.000	1.000	2314	tags=35%, list=24%, signal=46%
18	HALLMARK_CHOLESTEROL_HOMEOSTASIS	Details ...	58	0.22	0.84	0.750	0.991	1.000	2131	tags=26%, list=22%, signal=33%
19	HALLMARK_BILE_ACID_METABOLISM	Details ...	67	0.20	0.78	0.846	1.000	1.000	2520	tags=27%, list=26%, signal=36%
20	HALLMARK_FATTY_ACID_METABOLISM	Details ...	127	0.17	0.74	0.947	1.000	1.000	1629	tags=17%, list=17%, signal=20%
21	HALLMARK_TGF_BETA_SIGNALING		35	0.19	0.68	0.926	1.000	1.000	2677	tags=31%, list=28%, signal=43%
22	HALLMARK_XENOBIOTIC_METABOLISM		146	0.14	0.63	0.999	1.000	1.000	1766	tags=16%, list=18%, signal=19%
23	HALLMARK_ADIPOGENESIS		165	0.11	0.49	1.000	0.999	1.000	3765	tags=40%, list=39%, signal=64%

Table: Gene sets enriched in phenotype na ***[plain text format]***

	GS<br>follow link to MSigDB	GS DETAILS	SIZE	ES	NES	NOM p-val	FDR q-val	FWER p-val	RANK AT MAX	LEADING EDGE
1	HALLMARK_INTERFERON_GAMMA_RESPONSE	Details ...	158	-0.57	-2.99	0.000	0.000	0.000	2762	tags=73%, list=29%, signal=100%
2	HALLMARK_INTERFERON_ALPHA_RESPONSE	Details ...	84	-0.61	-2.90	0.000	0.000	0.000	2432	tags=74%, list=25%, signal=98%
3	HALLMARK_INFLAMMATORY_RESPONSE	Details ...	87	-0.58	-2.70	0.000	0.000	0.000	2638	tags=68%, list=27%, signal=92%
4	HALLMARK_ALLOGRAFT_REJECTION	Details ...	116	-0.54	-2.65	0.000	0.000	0.000	2541	tags=57%, list=26%, signal=76%
5	HALLMARK_COMPLEMENT	Details ...	161	-0.49	-2.50	0.000	0.000	0.000	2555	tags=50%, list=26%, signal=66%
6	HALLMARK_APICAL_JUNCTION	Details ...	146	-0.44	-2.25	0.000	0.000	0.000	2556	tags=47%, list=26%, signal=62%
7	HALLMARK_KRAS_SIGNALING_DN	Details ...	75	-0.47	-2.17	0.000	0.000	0.000	2134	tags=45%, list=22%, signal=58%
8	HALLMARK_IL6_JAK_STAT3_SIGNALING	Details ...	43	-0.48	-1.98	0.000	0.000	0.001	2677	tags=56%, list=28%, signal=77%
9	HALLMARK_COAGULATION	Details ...	119	-0.38	-1.91	0.000	0.001	0.004	2762	tags=40%, list=29%, signal=56%
10	HALLMARK_P53_PATHWAY	Details ...	135	-0.37	-1.91	0.000	0.001	0.004	2519	tags=44%, list=26%, signal=58%
11	HALLMARK_KRAS_SIGNALING_UP	Details ...	117	-0.37	-1.87	0.000	0.001	0.007	2553	tags=47%, list=26%, signal=63%
12	HALLMARK_TNFA_SIGNALING_VIA_NFKB	Details ...	107	-0.38	-1.85	0.000	0.001	0.008	2491	tags=45%, list=26%, signal=60%
13	HALLMARK_IL2_STAT5_SIGNALING	Details ...	117	-0.37	-1.84	0.000	0.002	0.011	1683	tags=29%, list=17%, signal=35%
14	HALLMARK_ANGIOGENESIS	Details ...	29	-0.46	-1.70	0.005	0.005	0.035	2354	tags=41%, list=24%, signal=55%
15	HALLMARK_PI3K_AKT_MTOR_SIGNALING	Details ...	83	-0.35	-1.63	0.000	0.008	0.056	1098	tags=24%, list=11%, signal=27%
16	HALLMARK_ESTROGEN_RESPONSE_LATE	Details ...	132	-0.32	-1.63	0.000	0.008	0.059	2541	tags=42%, list=26%, signal=57%
17	HALLMARK_MYOGENESIS	Details ...	164	-0.29	-1.53	0.000	0.017	0.135	4000	tags=52%, list=41%, signal=87%
18	HALLMARK_APOPTOSIS	Details ...	120	-0.29	-1.48	0.008	0.024	0.190	2335	tags=38%, list=24%, signal=50%
19	HALLMARK_PROTEIN_SECRETION	Details ...	94	-0.29	-1.40	0.018	0.043	0.322	2655	tags=36%, list=27%, signal=49%
20	HALLMARK_APICAL_SURFACE	Details ...	27	-0.32	-1.18	0.219	0.206	0.877	2002	tags=33%, list=21%, signal=42%
21	HALLMARK_ESTROGEN_RESPONSE_EARLY		129	-0.23	-1.15	0.163	0.230	0.912	2685	tags=39%, list=28%, signal=53%
22	HALLMARK_EPITHELIAL_MESENCHYMAL_TRANSITION		165	-0.22	-1.14	0.134	0.236	0.932	3392	tags=43%, list=35%, signal=65%
23	HALLMARK_HYPOXIA		141	-0.21	-1.09	0.241	0.305	0.974	1965	tags=24%, list=20%, signal=30%
24	HALLMARK_UV_RESPONSE_DN		110	-0.18	-0.89	0.768	0.822	1.000	3201	tags=43%, list=33%, signal=63%
25	HALLMARK_HEME_METABOLISM		142	-0.17	-0.88	0.824	0.819	1.000	2820	tags=33%, list=29%, signal=46%
26	HALLMARK_ANDROGEN_RESPONSE		71	-0.16	-0.76	0.960	0.973	1.000	1991	tags=21%, list=21%, signal=26%
27	HALLMARK_OXIDATIVE_PHOSPHORYLATION		195	-0.10	-0.54	1.000	0.998	1.000	1871	tags=12%, list=19%, signal=15%

Supplementary Figure 9. Correlations between SMYD3 and cell cycle- or EMT-related genes in the TCGA (mRNA levels) (A) and CPTAC (protein levels) (B) in the respective datasets of HPV-negative HNSCC cohorts. Correlation co-efficients R and p-values shown.

(A) TCGA

Cell cycle-related genes

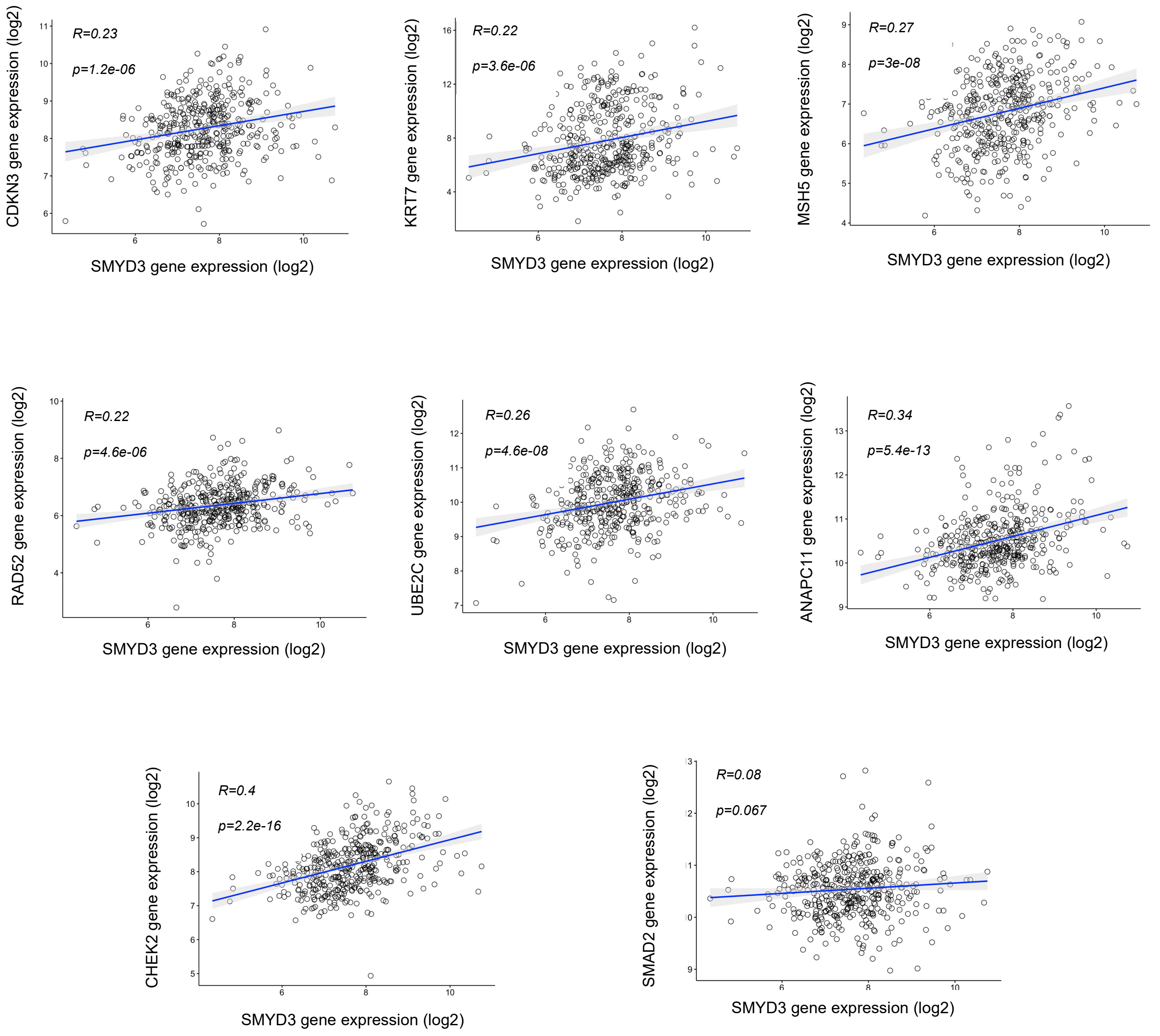

EMT-related genes

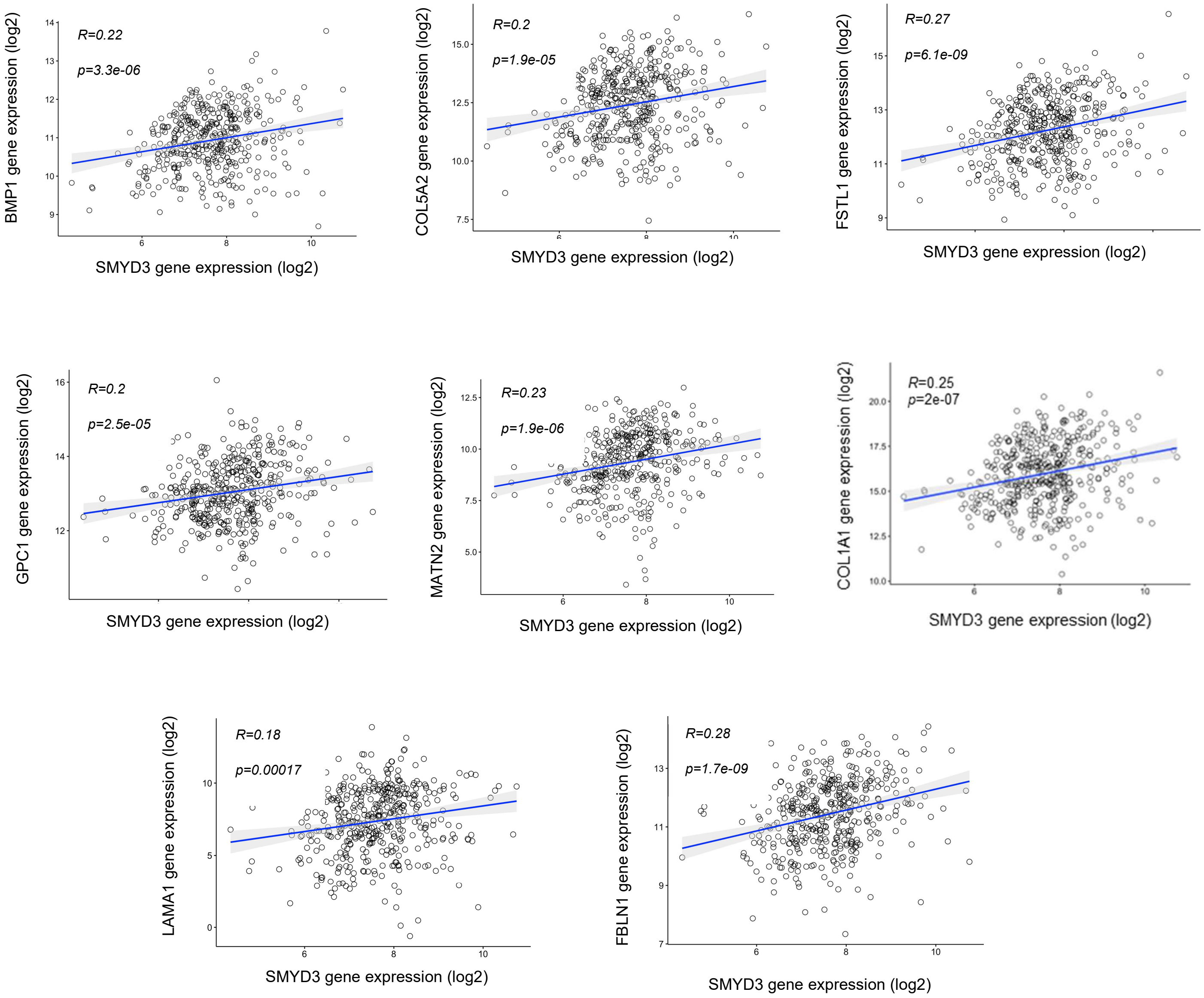

(B) CPTAC

Cell cycle-related genes

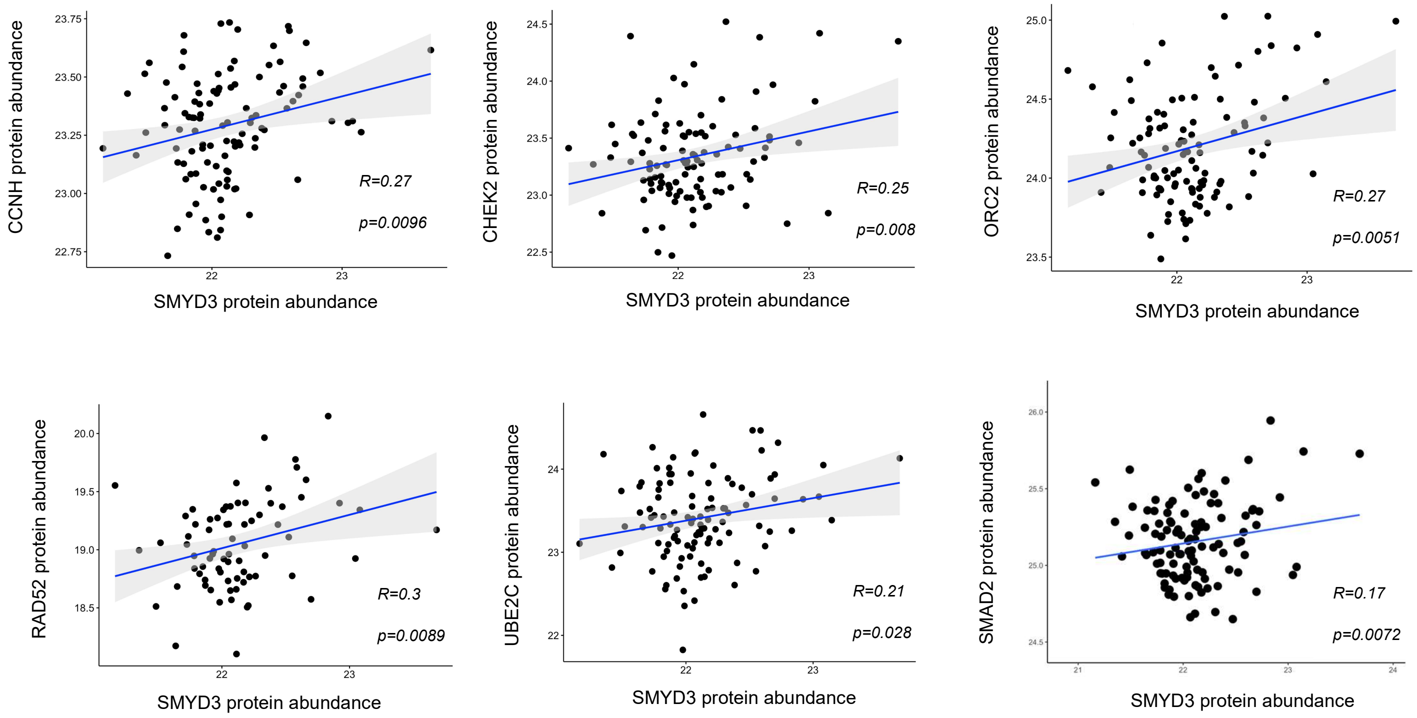

EMT-related genes

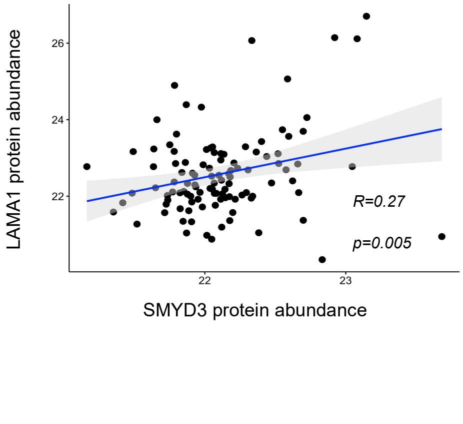

Supplementary Figure 10. Survival Kaplan-Meier curves based on SMYD3 mRNA and protein levels in HPV-negative HNSCC patients. Three independent cohorts of HPV-negative HNSCC patients were utilized (TCGA, University of Chicago cohort, CPTAC). Kaplan-Meier curves for progression-free survival **(A)**, and for overall (top) and progression-free survival (bottom) **(B, C)** based on SMYD3 expression levels. **(A)** TCGA HPV-negative HNSCC database (n=483). **(B)** The University of Chicago cohort (n=39). SMYD3 protein levels were semi-quantitatively assessed and binned into three scores: IHC score 1, 2 and 3. **(C)** CPTAC HPV-negative HNSCC database (n=108). Log-rank test p-values are shown.

(A)

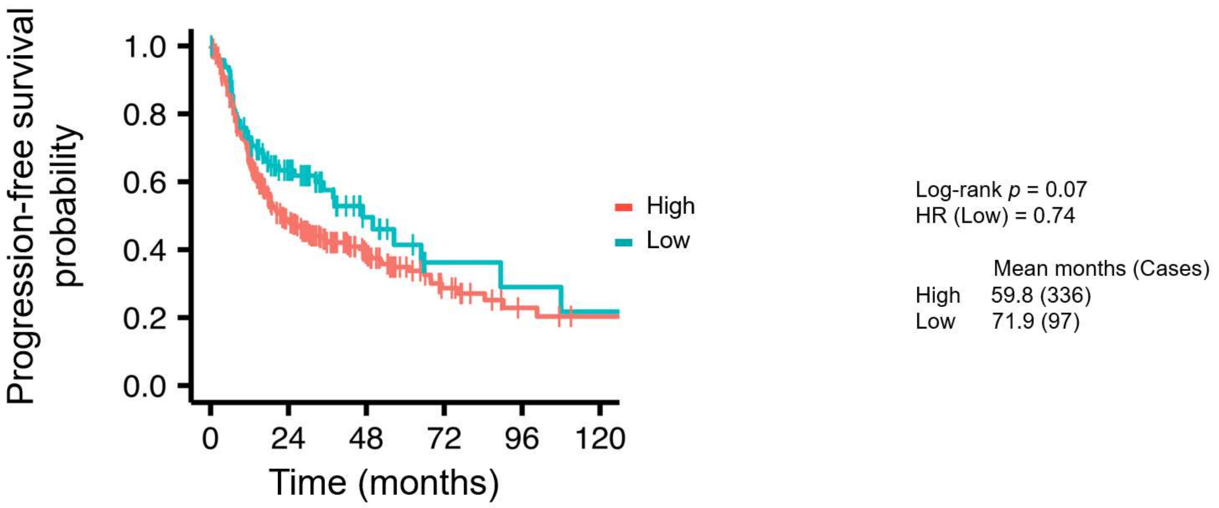

(B)

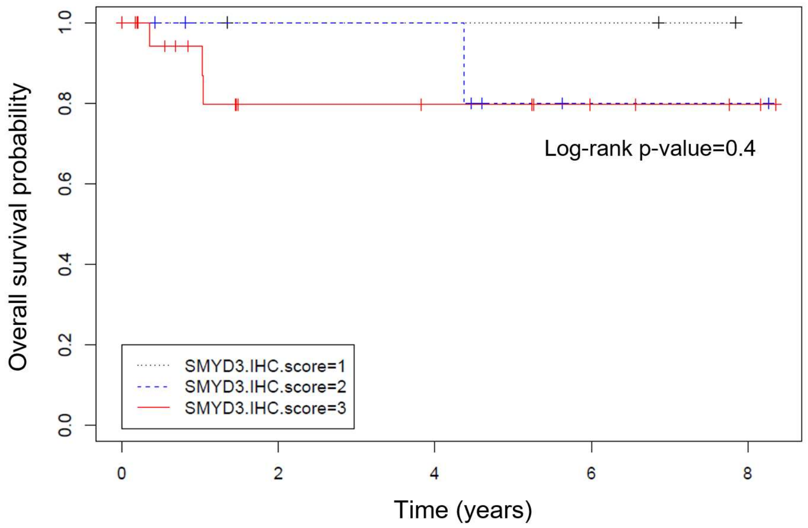

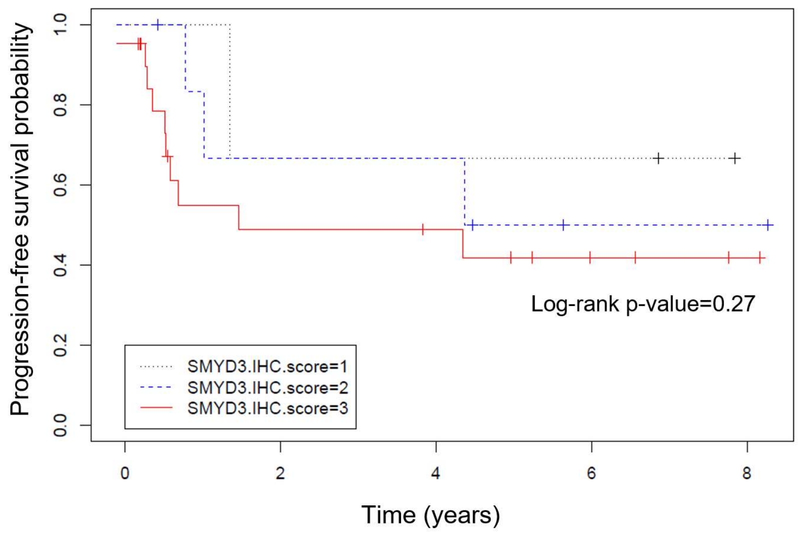
Progression-free survival probability
0.0
0.2
0.4
0.6
0.8
1.0
Log-rank p-value=0.27
SMYD3.IHC.score=1
SMYD3.IHC.score=2
SMYD3.IHC.score=3
0
2
4
6
8
Time (years)

(C)

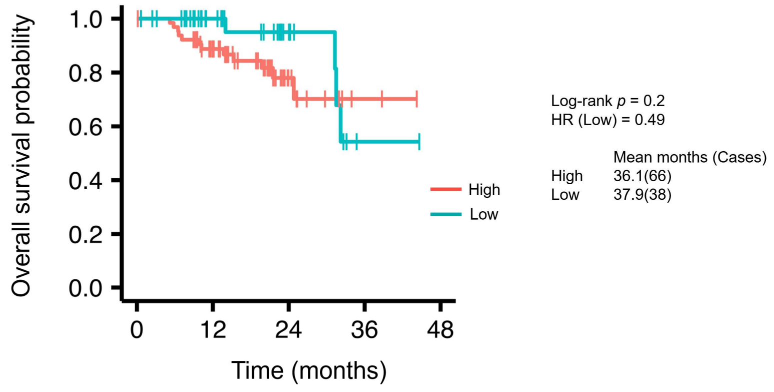
Overall survival probability
1.0
0.8
0.6
0.4
0.2
0.0
0
12
24
36
48
Time (months)
High
Low
Log-rank p = 0.2
HR (Low) = 0.49
Mean months (Cases)
High 36.1(66)
Low 37.9(38)

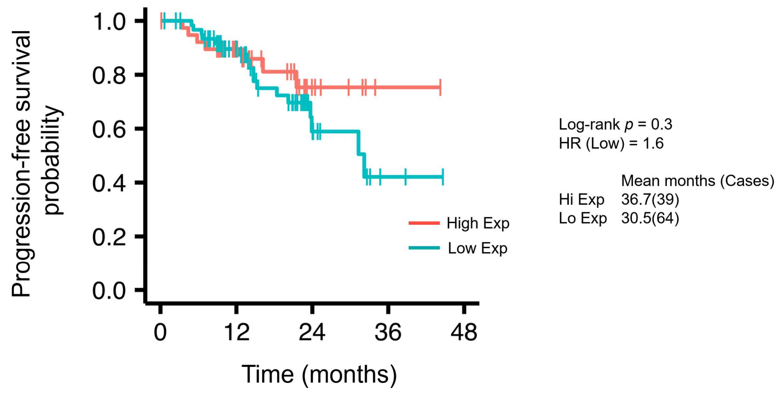
Progression-free survival probability
1.0
0.8
0.6
0.4
0.2
0.0
0
12
24
36
48
Time (months)
High Exp
Low Exp
Log-rank p = 0.3
HR (Low) = 1.6
Mean months (Cases)
Hi Exp 36.7(39)
Lo Exp 30.5(64)
